# Motif composition, conservation and condition-specificity of single and alternative transcription start sites in the *Drosophila *genome

**DOI:** 10.1186/gb-2009-10-7-r73

**Published:** 2009-07-09

**Authors:** Elizabeth A Rach, Hsiang-Yu Yuan, William H Majoros, Pavel Tomancak, Uwe Ohler

**Affiliations:** 1 Program in Computational Biology and Bioinformatics, Duke University, Science Drive, Durham, NC 27708, USA; 2 Institute for Genome Sciences and Policy, Duke University, Science Drive, Durham, NC 27708, USA; 3 Max Planck Institute of Molecular Cell Biology and Genetics, Pfotenhauerstrasse, Dresden 01307, Germany; 4 Department of Biostatistics and Bioinformatics, Duke University, Duke University School of Medicine, Erwin Road, Durham NC 27710, USA; 5 Department of Computer Science, Duke University, Durham, NC 27708, USA

## Abstract

**Background:**

Transcription initiation is a key component in the regulation of gene expression. mRNA 5' full-length sequencing techniques have enhanced our understanding of mammalian transcription start sites (TSSs), revealing different initiation patterns on a genomic scale.

**Results:**

To identify TSSs in *Drosophila melanogaster*, we applied a hierarchical clustering strategy on available 5' expressed sequence tags (ESTs) and identified a high quality set of 5,665 TSSs for approximately 4,000 genes. We distinguished two initiation patterns: 'peaked' TSSs, and 'broad' TSS cluster groups. Peaked promoters were found to contain location-specific sequence elements; conversely, broad promoters were associated with non-location-specific elements. In alignments across other *Drosophila *genomes, conservation levels of sequence elements exceeded 90% within the *melanogaster *subgroup, but dropped considerably for distal species. Elements in broad promoters had lower levels of conservation than those in peaked promoters. When characterizing the distributions of ESTs, 64% of TSSs showed distinct associations to one out of eight different spatiotemporal conditions. Available whole-genome tiling array time series data revealed different temporal patterns of embryonic activity across the majority of genes with distinct alternative promoters. Many genes with maternally inherited transcripts were found to have alternative promoters utilized later in development. Core promoters of maternally inherited transcripts showed differences in motif composition compared to zygotically active promoters.

**Conclusions:**

Our study provides a comprehensive map of *Drosophila *TSSs and the conditions under which they are utilized. Distinct differences in motif associations with initiation pattern and spatiotemporal utilization illustrate the complex regulatory code of transcription initiation.

## Abstract 2

A map of transcription start sites across the *Drosophila* genome, providing insights into initiation patterns and spatiotemporal conditions.

## Background

Transcription is a crucial part of gene expression that involves complex interactions of *cis*-regulatory sequence elements and trans-factors. It is mediated in large part through the binding of transcription factors (TFs) to DNA sequence motifs. The majority of eukaryotic genes (protein-coding genes and many regulatory RNAs) are transcribed by RNA polymerase II (RNA pol II), an enzyme that contains various subunits and can exist in a holoenzyme complex with several basal TFs, including TFIIB and TFIIF [1]. As RNA pol II does not have a direct affinity for the DNA, general TFs that bind to sequence motifs in the 100-bp region immediately surrounding the transcription start site (TSS), called the core promoter, guide it to the site of transcription initiation [2-4]. The set of general TFs includes TFIID, which consists of the TATA-box binding protein (TBP) and 10 to 14 TBP-associated factors (TAFs), along with TFIIH, and others.

Recent high throughput sequencing efforts based on 5' capping protocols have now generated capped transcripts for human and mouse on a high throughput scale under numerous conditions [5-7]. These '5'-capped' or 'cap-trapped' transcripts have helped to identify genomic TSS locations for thousands of genes, in particular for human, mouse and yeast [8-10]. This approach revealed that transcription is often initiated across widespread genomic locations, making it non-trivial to define initiation sites [5,7-11]. Two general initiation patterns have been characterized in mammalian core promoters. The first contains those with tags mapping to a 'single dominant peak,' whose promoters have strong over-representations of canonical motifs, such as the TATA box, GC box, CCAAT motif, and comparatively low frequencies of CpG islands. Gene Ontology (GO) analyses have shown that single dominant peaks are associated with developmental regulation and specialized differentiation processes [12]. The second type of initiation pattern comprises 'broad regions' whose promoters have TATA-poor profiles and are enriched in CpG islands. Broad regions are associated with more ubiquitously expressed transcripts with housekeeping functions, such as RNA processing and the ubiquitin cycle [12]. The large scale of available data allows for detailed analyses; for instance, one study explored the importance of precise spacing between the TATA box and the TSS [13].

Until recently, data comparable in scope to the capped analysis of gene expression (CAGE) sets for mouse and human have not been available for *Drosophila *genomes [14,15], but a large number of expressed sequence tags (ESTs) generated from different conditions have been sequenced in *D. melanogaster *using 5' capping technology [16]. Using these, several computational efforts have focused on the locations and frequencies of sequence motifs found in core promoters. The TATA box (TATA), initiator (INR), downstream core promoter element (DPE), and motif ten element (MTE) have been identified with distinct spacing requirements relative to the TSS [17]. Each of these motifs has been found at a comparatively low frequency, but several analyses have identified common additional motifs enriched in core promoters [18,19]. GO and microarray analyses have proved valuable in associating individual sequence elements with various functional terms, such as germline expression, and the embryo and adult stages of the fruit fly life cycle [19]. A different analysis showed that specific motif combinations, or modules, frequently occur in core promoters [20]. These modules are hallmarks of distinct core promoter types, and have been shown in a study of genes associated with highly conserved non-coding elements to characterize three main functional classes of genes in *D. melanogaster*: developmental regulation, housekeeping, and tissue-specific differentiation [21]. Such functional classes have also been associated with different modes of RNA pol II occupancy [22].

The core promoter elements and modules also offer deeper insight into the higher level organization of core promoter architecture. Genomic analyses are increasingly complemented by the elucidation of epigenetic patterns, such as the positioning of nucleosomes and the presence of certain histone marks [23,24]. Previous analyses used polytene chromosome staining and chromatin immunoprecipitation (ChIP)-on-chip to show the existence of two distinct transcriptional programs in *D. melanogaster*: TBP-related factor 2 (TRF2) regulation of TATA-less transcription, including the genes encoding linker histone H1; and TBP-regulated transcription, including transcription of promoters of the core histones H2A/B, and H3/H4 [25]. However, the degree to which the core promoter motifs/modules and epigenetic features are correlated with the patterns of transcription initiation and their usage during the stages of embryogenesis has not yet been explored in *D. melanogaster*.

In addition to the variability of initiation observed at a small scale at many individual start sites, a wide range of animal genes also possess clearly separated alternative promoters that are associated with specific functional consequences [26]. The extent to which such condition-specific variability is reflected in mammalian and *Drosophila *core promoters is so far mostly unclear. Several well-known *D. melanogaster *genes are known to use well-separated alternative promoters under different conditions. For instance, the transcriptional activator Hunchback (*Hb*) has two isoforms with different maternal (distal promoter) and zygotic (proximal promoter) patterns of initiation [27,28]. Alcohol dehydrogenase (*Adh*) utilizes two promoters, one during embryonic development and the second in adulthood [29]. As the presence and levels of TFs vary across tissues and time periods, arrangements of binding sites with which the TFs associate in the promoter region should reflect, to a certain degree, the conditions under which a specific core promoter is utilized [30,31]. However, genome-wide expression studies are typically based on gene-wide probes located in the coding or 3' untranslated regions. As a result, expression patterns made on a whole gene basis, such as those in FlyAtlas [32], in various conditions [33], neglect differences in distinct transcript variants. Low-throughput studies using primer extension or 5'RACE (rapid amplification of 5' complementary DNA ends) to evaluate the utilization of promoters at a higher resolution have also been typically done under one condition. This has restricted possible conclusions about the condition-specific usage of alternative promoters. Recent studies on tissue-specific TAFs showed that the core machinery is remodeled in specific conditions [34,35]. It is expected that the specificity of TAFs is encoded in additional core promoter sequence elements, although the sequence elements governing this regulation have been elusive.

In this work, we use available large-scale data to provide an extensive, high-quality mapping of alternative TSSs across the fruit fly genome. We show that core promoter elements and their corresponding modules are associated with peaked and broad patterns of transcription initiation. We also confirm that motif matches are highly conserved in the peaked promoters of TSSs, but show considerable variation in the broad promoters of TSS cluster groups. Next, we identify distinct associations of TSSs with spatiotemporal conditions based on the Shannon entropy of EST frequencies from different libraries. We investigate the specificity of alternative promoters at higher temporal resolution using available expression data from tiling arrays during embryonic development. Lastly, we identify intriguing trends of core promoter elements and their corresponding modules in maternally and zygotically utilized sites. Our analysis demonstrates that sequence elements in core promoters are directly associated with initiation patterns and the spatiotemporal conditions under which they are utilized.

## Results

### Identification and assessment of alternative start sites

#### EST clustering identifies a high-quality set of alternative transcription start sites

Previous studies on *Drosophila *promoters have often been based on the analysis of upstream sequences extracted from a genomic resource such as Flybase [36], using the most 5' location of a gene as the site of transcription initiation. However, using a resource in this way invariably leads to inconsistent assignment of TSS locations; for instance, many Flybase transcript annotations begin with a start codon, indicating that no transcript evidence is available and making the annotation incomplete on the 5' end. Filtering out such simple cases does not mean that the remaining transcripts are automatically 5' complete. While the accuracy of TSS annotations have considerably improved with increasing available data [37], the use of high throughput 5' capping methodologies to identify TSSs has also revealed dispersed patterns of transcription initiation in mammalian genomes [5,7]. These patterns have challenged the validity of choosing the most 5' observed location as being the consistently utilized site.

Thus, we are not confident in the reliability and quality of TSS data extracted from general-purpose genomic annotations because we cannot be sure which of the annotated 5' ends reflects a complete transcript, and which ones accurately capture a true and consistently used TSS. Other previous analyses in *D. melanogaster *were based on high quality TSSs, but were smaller in size and depth. For instance, our previous core promoter study covered 1,941 TSSs, but did not include alternative start sites [18]. The Eukaryotic Promoter Database (EPD) incorporates highly confident TSSs identified from the curation of ESTs and is of a similar magnitude to our previous study [38]. Here, we continue the tradition of using ESTs for TSS identification, but with the goal of identifying all of the consistently utilized and precisely defined TSSs, rather than the most 5' ones.

To minimize experimental error and clearly distinguish true TSSs from background noise, it is essential to filter available 5' transcript data. To accomplish this, we started from the large dataset of *D. melanogaster *ESTs in the Berkeley *Drosophila *Genome Collection (BDGC; Additional data file 1) [16,39]. A significant fraction of ESTs were obtained with a protocol designed at the RIKEN institute to capture capped full-length transcripts [9], similar to the more recent and larger mammalian efforts. This subset is therefore expected to map to the exact starting locations of known transcripts. While the amount of available ESTs is not large enough to completely saturate the transcriptome, it had until recently been the largest amount of transcript data for *Drosophila*. We mapped the BDGC ESTs derived from 15 different libraries to 8 distinct conditions: embryo, larva/pupa, head, ovary, testes, Schneider cells, mbn2 hemocytic cells, and fat body. A broad adult stage can be accounted for by combining the promoter associations of the head, ovary, testes, mbn2 hemocytic cell, and fat body. Additional libraries from more than one body part or time period, an unknown source, or additional conditions to those examined here were assigned to one default condition called 'diverse'. By using independently generated cDNA libraries, we expect to reduce potential experimental biases from any one library due to incomplete reverse transcription (Additional data file 1). This list of EST-library derived conditions is certainly limited, but it enables an initial analysis of promoter utilization in different life stages and differentiated tissues.

**Figure 1:**
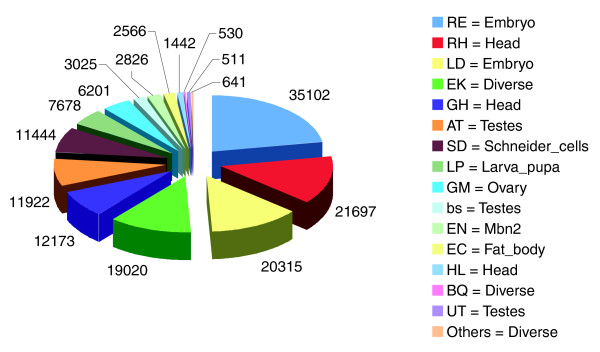
Sources of EST data. We took 631,239 EST alignments for 318,483 ESTs from the BDGC for release 4.3 of the fly genome annotation. The ESTs, derived from 16 main libraries, were filtered to a unique set of 157,093 alignments.

We started from a set of 631,239 EST alignments for 318,483 ESTs, which were part of release 4.3 of the *D. melanogaster *genome. We filtered this initial set to a reduced set of 157,093 unique EST alignments with high confidence of mapping to the 5' ends of transcripts (see Materials and methods). These unique EST alignments map across the *Drosophila *chromosomes and were derived from libraries of different sizes and conditions (Figure 1). The libraries providing the most ESTs were the RIKEN Embryo, with 35,102 ESTs, and RIKEN Head, with 21,697 ESTs. The remaining 100,294 ESTs were collected from non-cap trapping libraries. On account of the large size of the RIKEN libraries, the embryo and head conditions contained the largest number of ESTs, 55,417 and 35,312, respectively. ESTs mapping to the diverse condition and those from the testes were next in size, followed by the Schneider cells, larva/pupa, and ovary. The mbn2 hemocytic cells and fat body conditions had the smallest numbers of ESTs.

#### Alternative transcription start sites are a widespread phenomenon in the fly genome

**Figure 2:**
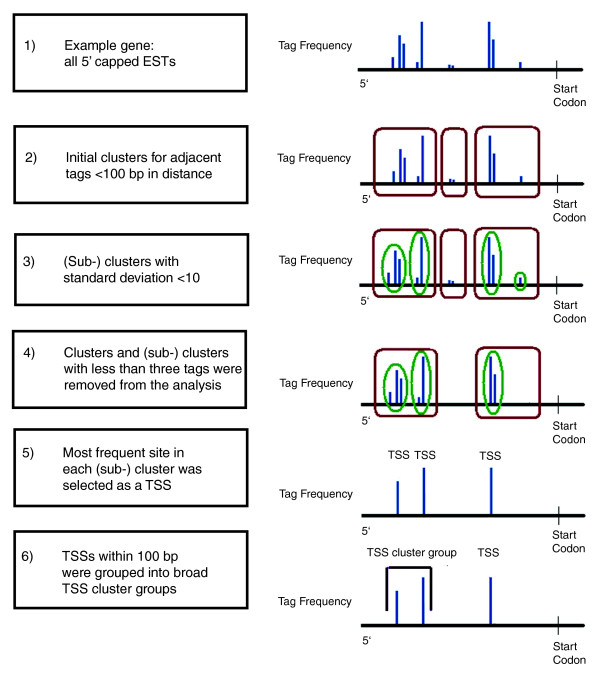
Hierarchical clustering algorithm and TSS identification. ESTs were hierarchically clustered in four main steps. 1) ESTs were mapped to the 5' ends of genes. 2) Large initial clusters were formed from grouping adjacent ESTs together that were less than 100 bp apart. 3) Clusters were broken into smaller (sub-) clusters that each had a standard deviation of less than 10. 4) (Sub-)clusters with less than three ESTs were removed. Then, 5) the most highly utilized location per (sub-)cluster was selected as the TSS and 6) TSSs within 100 bp were grouped into broad TSS cluster groups.

To obtain a set of the most consistently utilized and precisely defined TSSs, rather than the most 5', we implemented a hierarchical clustering strategy to define individual TSSs, as summarized in Figure 2 (see Materials and methods; Additional data file 1). We first associated each of the 157,093 filtered ESTs to corresponding genes, and then analyzed the distribution of ESTs for disjoint subsets, denoted '(sub-)clusters'. We selected one or more TSSs from these (sub-)clusters for each gene using additional criteria (see Materials and methods). All (sub-)clusters with less than three ESTs were removed from the analysis, and the individual TSS locations were required to be supported by at least two ESTs.

We identified 5,665 TSSs for 3,990 genes (Additional data file 2), nearly three times the number of TSSs and twice as many genes as in our earlier study [18]. More than half of the filtered ESTs were removed in hierarchical clustering and TSS selection. The largest decrease in the number of ESTs during TSS selection was observed for the diverse category. This indicates that data from more variable sources show less consistent TSS locations compared to RIKEN cap-trapped data. TSS locations with overlapping core promoter sequences - that is, less than 100 bp from each other - were grouped into non-overlapping TSS cluster groups spanning longer promoter regions. Below, the TSSs in TSS cluster groups are analyzed on two levels: as sites of individual initiation locations, and together when evaluating broad promoters.

When TSS locations were considered individually, there were 2,765 genes (69%) with one TSS, and 1,225 genes (31%) with alternative TSS locations. The 1,225 genes with alternative TSS locations were evaluated according to the initiation patterns of their promoters, and for 685 genes (56%) the alternative TSS locations were in one broad promoter, while for 540 genes (44%) the alternative TSS locations were in alternative promoters of the peaked or broad type, or any combination thereof. Genes with alternative promoters were distributed across chromosomes 2L, 2R, 3L, 3R, and X (Figure S1 in Additional data file 1). There may be additional alternative initiation sites upstream or downstream of those listed here that were not considered due to a lack of EST support.

The mean genomic distance from TSSs to the most upstream start codon annotated in release 4.3 was 1,353 bp, with a median of 264 bp. This is 91 bp smaller than our previous estimate of 1,444 bp between TSS and start codon using chromosome 2R [18]. This difference is likely due to the earlier strategy of Ohler *et al*. using the most 5' ESTs to define sites of transcription initiation, rather than our use of the most highly utilized locations as TSSs. For genes with a consistent downstream start codon annotation, 141 TSSs were more than 10,000 bp upstream of the closest start codon. This observation of large distances between TSSs and their corresponding start codons agrees with high frequencies of large distances between TSSs and start codons found in *D. melanogaster *using tiling arrays [40]. Due to the clustering criteria, the minimal distance between two alternative TSSs was 20 bp, with the most common distance ranging from 25 to 35 bp. This is different from the more high-resolution definition of alternative TSSs that was employed in studies using high-throughput 5' cap trapping data [13]. As a result, canonical core promoter sequence elements that occur at precise distances from the TSS, such as the INR, TATA box or DPE, can be clearly assigned to individual promoters.

The maximum number of individual TSSs identified per gene was seven for the genes CG33113 (*Rtnl1*), CG14039 (*quick-to-court*), and CG11525 (*CycG*). Flybase listed three fewer alternative TSSs for *quick-to-court*, and four fewer for *CycG *in release 5.11 [36]. Seven transcript isoforms for *Rtnl1 *and *quick-to-court*, and three transcript isoforms for *CycG *are annotated for these genes. Whereas some of the TSSs of *CycG *and *quick-to-court *are close to each other and combined in cluster groups, all of the TSSs of *Rtnl1 *are well-separated peaked TSSs. Due to the stringent selection criteria we employed in the clustering strategy, genes with more than seven promoters may exist, but we found the most common range of alternative TSSs to be much lower.

Due to the definition of the TSS cluster groups, the minimal distance between TSSs in alternative TSS cluster groups is 101 bp, and the most common intra-cluster distance ranges from 101 to 199 bp. There were 55 TSS cluster groups separated by more than 10 kb. It is estimated that noncoding 5' and 3' DNA each comprise approximately 2 kb of intergenic sequence, and that intergenic distances increase with regulatory complexity [41]. Genes performing house-keeping functions, such as ribosomal constituents and general TFs, are commonly spaced in 4 to 5 kb segments of DNA. Genes with more complex roles, such as in embryonic development and/or pattern specification, take up 17 to 25 kb of DNA on average. This suggests that some of the alternative TSSs/cluster groups separated by large distances may experience more complex transcriptional regulation.

**Figure 3:**
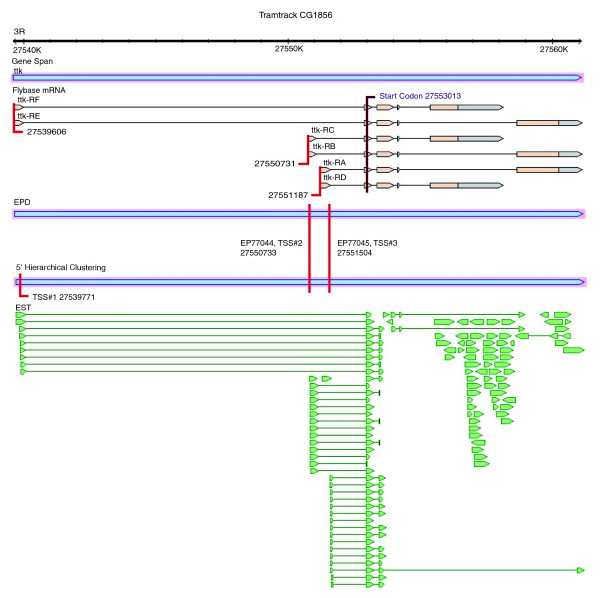
Alternative transcription start site annotation for the example gene *tramtrack*. Flybase annotation of TSSs at the *tramtrack *locus of telease 4.3 [36]. The gene span, Flybase mRNA, EST, and cDNA alignments were created using Gbrowse in Flybase [36]. The locations of the EPD sites, hierarchically clustered TSSs, and start codon were added manually. There were three peaked TSSs listed in Flybase at locations 27539606 (TSS#1), 27550731 (TSS#2), and 27551187 (TSS#3). A fourth site at position 27552854 was listed, and is not shown, as it corresponded to the first nucleotide of the exon containing the start codon across all transcripts, and is likely to be an annotation artifact. The first TSS in EPD, EP77044, is 2 bp downstream of the Flybase TSS#2 at location 27550733. The second TSS, EP77045, occurred at location 27551504, and is 317 bp downstream of Flybase TSS#3. The distributions of ESTs at both locations were classified as single initiation sites by EPD on account of their high frequency and small dispersion. In the hierarchically clustered set, we observed TSSs at locations 27539771 (TSS#1), 27550733 (TSS#2), and 27551504 (TSS#3). The two most downstream TSSs correspond to the TSSs in EPD, and the most upstream TSS is close to the first TSS annotated in Flybase, but missing in EPD. This agreement with EPD resulted from our use of a similar dataset and identification strategy. All three Flybase TSSs for *tramtrack *are upstream of TSSs in the EPD and our sets, highlighting the bias in the usage of the most 5' evidence as TSSs, rather than the most highly utilized locations. Looking at the presence of sequence motifs within *tramtrack *peaked promoters, an INR was present at both TSS#1 and TSS#3 as defined in our set, strengthening our assignments for these TSSs, in spite of their considerably different locations in Flybase.

We evaluated the quality of our set of alternative TSSs by comparing initiation locations and promoter composition of it to sites in the EPD and Flybase (Figure S2 in Additional data file 1). While EPD and Flybase provide high quality support for the identified sites across the *Drosophila *genome, for a single gene the TSS location information is often incomplete using either database, and inconsistent using both. The TSSs identified by hierarchical clustering thus supplement current annotations by providing precise and consistent TSS locations. We illustrate this for the gene *tramtrack *(*ttk*; CG1856), a transcriptional repressor located on chromosome 3R (Figure 3).

### Presence and conservation of core promoter motifs

#### Sequence elements are associated with different initiation patterns

For more than 20 years, it has been known that some promoters are highly position-specific, while others are spread over larger regions [42]. The analysis of large-scale CAGE data in mammals has confirmed the presence of peaked and broad promoters as a general phenomenon, and led to a more precise definition of four different promoter shapes reflecting different initiation patterns [12]: 1, single-peaked or focused; 2, broad or dispersed; 3, multimodal; and 4, broad with peak(s). In the clustering analysis above, we identified two types of promoters: 'peaked ' for single TSSs, and 'broad' for TSS cluster groups. The scale of the available fly data does not allow for a more precise sub-classification, but the two groups resemble the categories found in mammals to some extent, with the broad promoters being a potential combination of categories 2 to 4.

Compared to mammals, analyses of the *Drosophila *genome have identified a larger set of sequence motifs enriched in core promoters. Ohler *et al*. [18] predicted a set of ten motifs in the [-60,+40] bp region surrounding the TSS; Fitzgerald *et al*. [19] later identified 13 motifs with enrichment in the same region, including nine of the ten motifs from Ohler *et al*. This knowledge allowed us to investigate whether the peaked and broad promoters were associated with specific core promoter elements, similar to the TATA box and CpG island biases found in mammals [12]. We focused on eight of the ten motifs in Ohler *et al*. that have either been biologically validated or previously reported as building blocks for core promoter sequence modules. The eight motifs included four location-specific canonical motifs (TATA, INR, DPE, and MTE) [43], and four motifs that have weaker positional biases, but were found to frequently co-occur in a specific order and orientation (Ohler 1, DNA replication element (DRE), Ohler 6, and Ohler 7) [19,20]. Of the latter, only the role of the DRE in the recruitment of the polymerase has been unraveled [44]. We evaluated the occurrence of these eight motifs and their most frequently occurring modules in 3,788 peaked and 876 broad promoters (see Materials and methods). Because there were far more peaked promoters than broad promoters, their core promoters covered a three times larger genomic region. To provide an equal measure across both sets, and across motifs with differences in location preferences, motif matches were counted anywhere in the promoters, and the numbers of motifs found were then normalized to the number of occurrences per 100 kb. For an estimation of the numbers of motif frequencies expected by chance, the analysis was repeated on three sets of 100-bp regions surrounding randomly selected intergenic sites.

**Figure 4:**
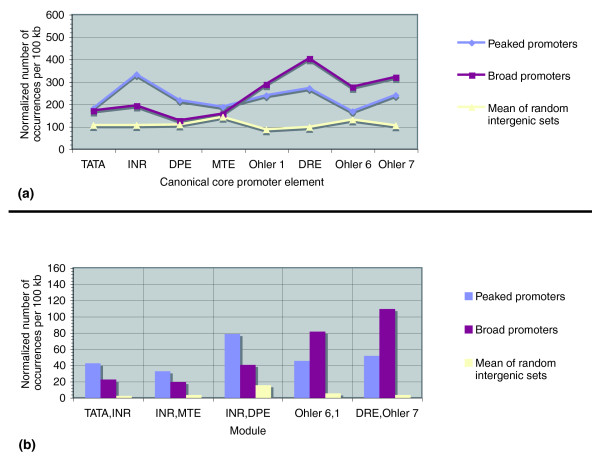
Core promoter elements are associated with initiation pattern. PATSER was used to evaluate the presence of the eight core promoter elements at any location in the 100-bp sequences surrounding 3,788 TSSs, 876 TSS cluster groups, and three sets of 1,299 random intergenic sites. All counts were rounded to the nearest whole number after normalization. **(a) **Individual motif occurrences. The number of motif matches were counted and normalized to the number of occurrences per 100 kb. For the random intergenic sites, the mean numbers of motif occurrences across all three sets are shown. **(b) **Module occurrences. The number of pairs of motif matches present in the designated order, with respect to the orientation of transcription, were counted and normalized to the number of occurrences per 100 kb.

Figure 4a shows a clear separation in core element usage between peaked and broad promoters. While the TATA, INR, DPE, and MTE were more prevalent in peaked promoters, broad promoters had larger numbers of the Ohler 1, DRE, Ohler 6 and Ohler 7. As the TATA, INR, DPE, and MTE occur more frequently at specific locations from the site of initiation, and the Ohler 1, DRE, Ohler 6 and Ohler 7 have a weaker positional bias, peaked and broad initiation patterns directly correspond to the strength of location biases of the promoter elements that define them. With the exception of the INR, there were fewer occurrences of the location-specific canonical elements in peaked promoters than there were of the motifs without location bias in the broad promoters. As this relationship appears after normalization, this suggests that the density of motifs is not linearly proportional to the genomic span of the core promoters, but rather that broad promoters, which include multiple closely spaced initiation sites, also contain higher densities of their most frequent elements.

The greatest difference in element frequency between peaked and broad promoters was observed for the INR and DRE. This suggests that the DRE may be of equal importance to transcription for broad promoters as the INR is for the peaked promoters. All motif observations were higher than the mean number of occurrences found across the three random intergenic sets, and random occurrence rates corresponded well to the expectation based on motif score cutoffs. When motifs in peaked promoters were constrained to their functional locations (see Materials and methods), the same trends of occurrences were observed (Figure S3a in Additional data file 1). We did not analyze restricted motif locations for the broad promoters, as multiple TSS reference points in the TSS cluster groups prevented distinct assignments within the overlapping core promoters.

Next, we evaluated the presence of combinations, or modules, of known elements in the core promoters of the peaked TSSs and broad TSS cluster groups. A previous study had identified five different core promoter modules, which we evaluated here: TATA/INR, INR/MTE, INR/DPE, Ohler 6/1, and Ohler 7/DRE [20] (see Materials and methods; Additional data file 1). Figure 4b shows that the TATA/INR, INR/MTE, and INR/DPE modules occurred more frequently in the peaked promoters, and the Ohler 6/1 and Ohler 7/DRE modules were more prevalent in the broad promoters. This corresponds with our results of the occurrences of the individual elements. It also shows that even though the Ohler 6 and Ohler 7 elements have a lower positional bias, they occur in a specific order within binding modules. All module occurrences in peaked and broad promoters were far above the mean number found in the three random intergenic sets, although higher numbers of the most frequent modules appeared in the broad promoters than in those of peaked promoters. This reaffirms that the broad core promoters of TSS cluster groups have a higher density of the most frequent modules of motifs than those of individual TSSs. Extending the analysis to three elements is limited by the rareness of such events, but analyses indicated that INR/MTE/DPE and TATA/INR/DPE occurred more often than triplets of elements with less positional bias (data not shown).

Finally, peaked core promoters were found to have higher frequencies of G (0.229) and C (0.234) than broad core promoters (G, 0.211; C, 0.224) and the 100-bp sequences surrounding the random intergenic sites (G, 0.203; C, 0.205). These results confirm previous work showing that core promoters with the DPE, INR, and TATA/INR have a moderate GC content, and core promoters with the DRE, and Ohler 1/6 elements have a GC-poor profile [20]. With this analysis, we show that the GC content is not only characteristic of core promoter elements, but also of initiation patterns of transcription.

#### Conservation of sequence elements differs across initiation patterns

**Figure 5:**
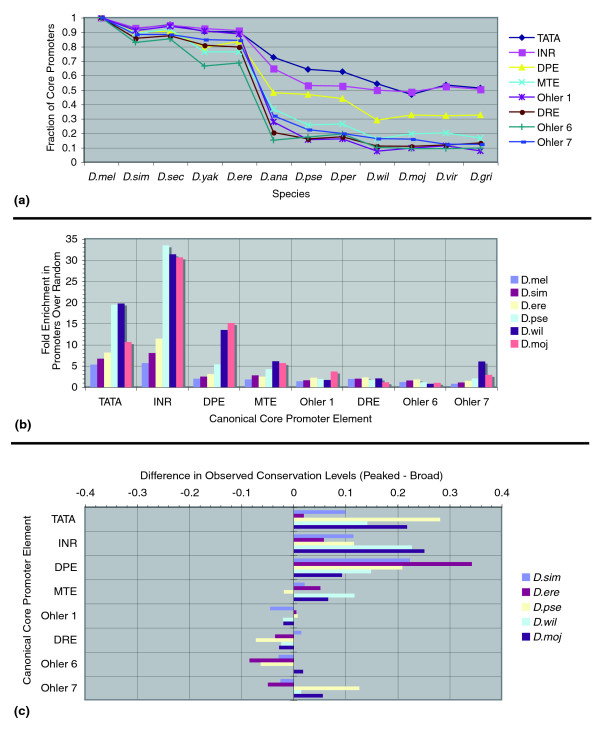
Evolutionary conservation of sequence elements. The core promoter sequences surrounding each *D. melanogaster *TSS were mapped to orthologous locations in the 12 *Drosophila *genomes. **(a) **Conservation of sequence elements across the 12 fruit fly genomes. The set of *D. melanogaster *promoters having an element present in its preferred window was selected, and the fraction of all orthologous sequences with the motif present was assessed in a pairwise fashion with the other 11 species. The figure indicates a sharp decline in the conservation of the elements outside of the *melanogaster *subgroup. **(b) **Enrichment of conserved motif matches in promoters over random sequences. The plot shows the fold enrichment of the fraction of total *D. melanogaster *motif matches conserved in the preferred window of 100-bp sequences surrounding detected TSSs compared to random intergenic locations. For clarity, the plot shows only five out of the eleven species in the total pairwise comparisons. **(c) **Differences in conservation of canonical elements between peaked versus broad promoters. After splitting the motif matches used in (a) by their occurrence in peaked versus broad promoters, there are noticeable differences between the conservation levels of motifs. For clarity, we again only show five out of the eleven pairwise species comparisons. *D. mel*, *D. melanogaster*; *D. sim*, *D. simulans*; *D. sec*, *D. sechellia*; *D. yak*, *D. yakuba*; *D. ere*, *D. erecta*; *D. ana*, *D. ananassae*; *D. pse*, *D. pseudoobscura*; *D. per*, *D. persimilis*; *D. wil*, *D. willistoni*; *D. moj*, *D. mojavensis*; *D. vir*, *D. virilis*; *D. gri*, *D. grimshawi*.

Given the different associations of motifs with initiation patterns, we sought to examine whether there were differences in the conservation of core promoter motifs across the 12 fully sequenced *Drosophila *genomes. We selected the promoters of individual TSSs and TSSs in TSS cluster groups that had aligned sequences in all 12 species (see Materials and methods). This led to a reduced set of 4,243 promoters for 3,175 genes: 2,886 peaked TSSs, and 1,357 TSSs in broad promoters. We compared the conservation of the eight core promoter motifs in *D. melanogaster *to the other eleven genomes in a pairwise fashion (see Materials and methods). In other words, we assessed whether a presumably functional motif, defined by the occurrence of a motif match in the preferred window relative to the location of a mapped TSS in *D. melanogaster*, was still detected in a second species in the corresponding position in the alignment. Figure 5a shows that conservation levels of the INR motif ranged from approximately 90 to 95% for promoters in the *melanogaster *subgroup to approximately 50% for promoters in distantly related species. These levels directly correlate with the phylogenetic distances of the 12 genomes [14]. Similar patterns are found for the other position-specific motifs, with the TATA box showing the highest level of conservation, and the MTE the lowest in more distant species. For the other four motifs, the conservation levels were consistently lower.

While this analysis showed clear trends, it did not indicate whether such observations could arise from chance. We therefore determined the fraction of pairwise conserved motif matches by dividing the number of conserved motif instances in the preferred window over the total number of occurrences anywhere in the *D. melanogaster *promoters. After repeating this analysis on a set of similar sized random intergenic sequences, we took the ratio between promoters and random sequences as the motif enrichment score; for *D. melanogaster *alone, this score simply indicated the enrichment of hits in the preferred window (Figure 5b). In general, ratios were higher for the position-specific motifs INR, TATA, MTE, and DPE, with the INR exceeding enrichments of 30-fold. While there was a lower but consistent score for Ohler 1 and DRE, the motifs Ohler 6 and Ohler 7 did not clearly exceed a ratio of 1 in *D. melanogaster*, indicating that the preferred windows taken from [19] were not actually enriched above background. The total number of conserved instances was quite low for these motifs, and the higher scores seen for more distantly related species may be regarded with caution, as they could simply be a side effect of the small sample size. Nonetheless, we saw that the motifs that were less restricted in their relative location to the TSS showed a lower level of conservation in the aligned locations.

Given that these two motif sets were shown to be associated with different initiation patterns, we assessed whether motifs in peaked promoters exhibited different conservation patterns than those in broad promoters. Figure 5c shows that there are indeed strong differences in the conservation levels of motifs across initiation patterns. Conservation levels of localized motifs (TATA, INR, DPE, MTE) were consistently higher when they occurred at peaked TSSs versus TSSs in broad promoters. This trend was mirrored in a somewhat weaker fashion by the set of motifs with lower positional preference (Ohler 1, DRE, Ohler 6, Ohler 7), which were more conserved in peaked than broad promoters. Observations on promoter conservation and TSS turnover have been reported for human-mouse comparisons supported by 5' capped tag data [45]. In particular, findings indicated that some alternative promoters experience a lower negative selective pressure, and this may reflect an intermediary stage of a TSS turnover event. Our findings here indicate that selective pressure on the motifs in promoters also depends on the initiation patterns, with evidence that broad promoters may experience more frequent functional motif turnover due to the lowered restrictions on relative spacing of enriched motifs, and/or the presence of other functional promoters in the close vicinity.

Looking at the conservation of motifs for the *ttk *case study (Figure 3), we recall that two INR motifs were present in the preferred location of the peaked promoters of TSS#1 and TSS#3. The initiator motif in the TSS#1 promoter was conserved across all 12 species, and the initiator in the TSS#3 promoter was conserved within the 5 species of the *melanogaster *subgroup. This illustrates the existence of differences in motif occurrence and conservation levels at alternative start sites.

### Condition-specific utilization of promoters

#### Transcription start sites have distinct associations with conditions derived from EST libraries

Sites of transcription initiation are determined by the conditions under which transcription factors mediate the recruitment of RNA pol II to the core promoter. Associations of TSSs with conditions can give insight into the utilization and organization of TF binding sites in core promoters. For this reason, we characterized the condition associations of the set of 5,665 TSSs identified from (sub-)clusters in the hierarchical clustering of 5' ESTs in *D. melanogaster*, regardless of initiation pattern, into three groups (condition-specific, condition-supported, mixed) using Shannon entropy (see Materials and methods; Additional data file 1). As mentioned above, the cDNA library information for each of the ESTs was mapped to one of eight distinct conditions (embryo, larva/pupa, head, ovary, testes, Schneider cells, mbn2 hemocytic cells, and fat body) plus a default (diverse) category. Overall, the data are more descriptive of spatial body parts than of well-resolved temporal stages of *Drosophila *development.

**Figure 6:**
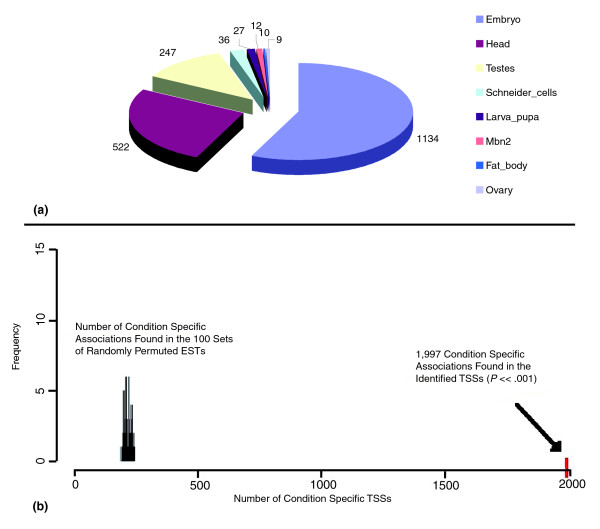
Condition-specific associations of TSSs as determined by Shannon entropy. **(a) **Condition associations for the set of identified TSSs. Shannon entropy was applied to 72,535 ESTs in the (sub-)clusters of 5,665 identified TSSs. There were 33,077 ESTs from embryo, 23,361 from head, 3,903 from Schneider cells, 2,883 from testes, 2,267 from larva pupa, 1,978 from ovary, 699 from mbn2 cells, 471 from fat body, and 3,896 with the diverse label. The degree of association of the TSSs with the spatiotemporal conditions was evaluated using EST frequency, Shannon entropy, and a tripartite classification system (see Materials and methods). The numbers of TSSs with specific associations are shown. **(b) **Condition associations for random permutations of labels. Condition assignments were repeated on 100 sets of random permutations of the 72,535 condition labels across the 5,665 (sub-)clusters. The total number of sites with specific condition associations was summed for each permutation. Across all 100 sets of permutations, the number of condition-specific sites ranged from 180 to 250. The 1,997 condition-specific TSSs in the identified set significantly deviated from this distribution (*P *<< 0.001).

There were 1,997 (35%) TSSs with specific associations (Figure 6a), and 1,612 (29%) TSSs with supported associations in one of the eight conditions (Additional data file 4). Together, almost two-thirds of the TSSs had associations with only one condition. Specific and supported assignments existed for TSSs across all conditions, with the embryo and the head having the largest numbers of specific or supported sites. The testes had the third largest number of specific TSSs (247), and the ovary had the smallest number of specific TSSs (9). The numbers of testes and ovary TSSs were comparatively higher than their fraction within the set of filtered ESTs. There were 14% of TSSs that were supported in two conditions. The two largest pairs of condition associations were embryo:head and embryo:Schneider cells. The embryo:head pair can be accounted for by the large sizes of the ESTs in their libraries, and the embryo:Schneider cell pair can be explained by the fact that Schneider cells are derived from embryos at 20 to 24 hours of development. There were 1,275 (22%) TSSs classified as having mixed associations. By default, we labeled TSSs that were specific or supported for the diverse condition as having mixed associations because their supporting ESTs were derived from broad or unknown conditions. The existence of library bias that can affect the determination of the condition specificity of the TSSs was taken into account (Additional data file 1). We evaluated the significance of the results and found that the number of 1,997 condition-specific TSSs was significantly higher than expected by random permutations (*P *<< 0.001; Figure 6b; Additional data file 1).

When considering condition associations on a gene level, the numbers of specific, supported, and mixed TSSs did not significantly differ for genes with alternative TSSs compared to those having single TSSs, indicating that the presence of condition associations for more than one core promoter is a common phenomenon across all conditions. Because we assigned conditions to individual TSSs, it was possible for the 1,225 genes with alternative TSSs to have more than one association. We thus divided genes with alternative TSSs into two groups: genes whose TSSs had different condition associations, if at least one TSS had at least one different association from the gene's remaining TSSs; and genes with the same condition associations for all of the alternative initiation sites. In our dataset, 392 (32%) genes with alternative TSSs had the same condition association, and over two times that number of genes with alternative TSSs (833; 68%), had different condition associations. The number of genes with different conditions was significantly lower than expected when evaluated using random permutations of the condition association labels (*P *<< 0.001; Additional data file 1). However, with additional conditions and ESTs, we expect to observe a larger percentage of alternative TSSs with different associations.

For the previously mentioned example gene *ttk*, all three TSSs had embryo associations. The two most upstream TSSs were embryo-supported, and the third downstream TSS was embryo-specific. The associations corresponded to the known expression of the gene during embryogenesis for various functions, including the regulation of proper development of tissues [46] and the determination of cell-fate [47]. This association of *ttk*'s TSSs exemplifies typical patterns seen for the set of 392 genes with alternative TSSs having the same condition associations. Additional examples of the EST condition associations confirming known expression patterns and developmental regulation of genes are provided in Additional data file 1. While these assignments do not determine function, they help to define the scope of alternative promoter utilization and contribute novel information about expression patterns.

#### Differences in the temporal utilization of alternative promoters during embryogenesis

While we observed a significant enrichment of alternative TSS associations with the same conditions, EST libraries are too broad to distinguish differences in the precise timing of a promoter's temporal utilization. To examine initiation events at higher resolution, we used available Affymetrix whole-genome tiling arrays of *D. melanogaster *embryonic expression. The data were a natural fit to our analysis because expression of genes was monitored at 12 time points during the first 24 hours of the developing *D. melanogaster *embryo, each covering a 2-hour period [40]. Embryogenesis has been well studied in *Drosophila*, and the morphological changes that occur have been examined in depth. The control of transcription initiation during early embryogenesis involves well-known TFs, such as Kruppel and Eve [2]. Their utilization has become an important model system for studying the complexity of gene regulation.

**Figure 7:**
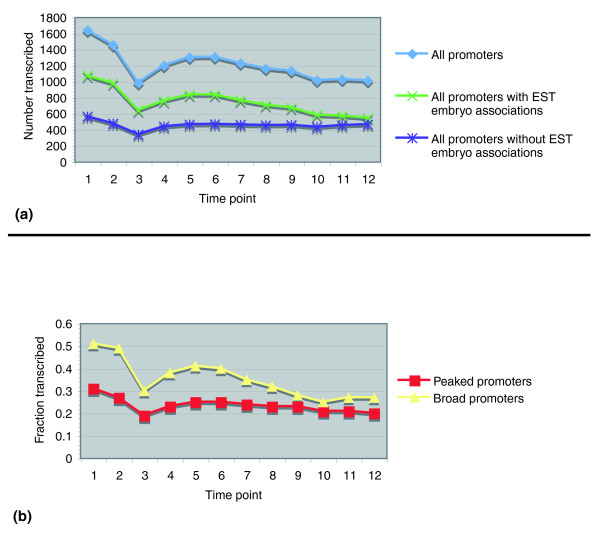
Embryonic utilization measured by Affymetrix tiling arrays. **(a) **Consistent trend of utilization across EST and tiling experiments. Median differences in tiling array fluorescence intensities were used to detect transcription at 4,664 peaked and broad promoters. The total number of transcribed sites was plotted for each of the 12 time points, corresponding to 2-hour increments during embryogenesis. The promoters were separated into two groups at each time point: those with embryo EST associations and those without. **(b) **Developmental condition is correlated with initiation patterns. The set of all promoters was divided into 3,788 peaked and 876 broad. At every time point, the fractions of transcribed peaked and broad promoters were found by dividing the number of transcribed promoters in each group by the total number of peaked and broad promoters, respectively.

Each of the oligos used in the array was 25 bp in length, spaced at approximately 35-bp intervals genome-wide. Unlike ESTs, which allowed us to assign TSS associations at the level of individual nucleotides, the limited tiling resolution restricted our ability to distinguish differences in transcriptional activity of promoters at individual TSSs. Therefore, we analyzed the temporal embryonic utilization of peaked promoters separated by more than 100 bp and broad promoters. We evaluated activity of 2,765 genes with one peaked promoter, 685 genes with one broad promoter, and 540 genes with a combination of promoter types (see Materials and methods; Additional data file 5). Our methodology resulted in a low expected false positive rate of 0.02 to.035 (Additional data file 1) and, by pooling all promoters together, we saw 58.7% transcribed in at least one of the 12 embryonic time points. The largest number of promoters (1,640 and 1,455, respectively) was utilized at time points 1 and 2, compared to any other developmental period (Figure 7a). These results agreed with previous analyses of the tiling data that focused on whole transcripts [40]. At this early stage in development, most promoters are expected to correspond to maternal utilization. There was a decrease in the number of promoters utilized at time point 3, followed by a second maximum of approximately 1,300 promoters utilized at time points 5 and 6. This corresponded to the decrease in maternally inherited transcripts and the initiation of zygotic transcription. After time point 6, the number of promoters utilized continued to decrease, with a third weaker maximum at period 11. The presence of these three cycles suggests periods during which the binding of TFs and/or RNA pol II differs for large numbers of genes during embryogenesis. Further statistical analysis is needed to rigorously evaluate the significance of this trend. Overall, 1,682 peaked and 288 broad promoters showed no utilization during any of the 12 developmental time points.

Temporal biases of transcriptional activity were seen in the tiling array when the total number of promoters was divided into peaked and broad. After normalization by the total number of promoters in each set, a statistically significant higher fraction of broad promoters was utilized than peaked promoters in the tiling array (*P *<< 0.01; Figure 7b; see Materials and methods). The difference was greatest in the first and second 2-hour periods, and reached an additional maximum at time points 5 and 11. While it continued to decrease after time point 5, the difference remained through time point 12. Overall, 56.6% of peaked promoters were transcribed in at least one of the 2-hour periods, and 67.8%, or 11.2% more, broad promoters were transcribed in at least one period. The pattern that broad promoters were more transcriptionally active during embryogenesis than peaked promoters was separately mirrored using the EST associations alone, without the tiling array data (*P *<< 0.01; see Materials and methods). Here, initiation sites were deemed to have an embryo EST association if an individual TSS, or at least one of the TSSs in a TSS cluster group, had the association, resulting in 50.3% of TSSs and 74.3% of the TSS cluster groups having embryo-specific or embryo-supported associations. When comparing the condition associations of both promoter types across EST and tiling array experiments, we saw consistency in embryonic utilization of promoters (Figure 7a; Additional data file 1).

Finally, the time course tiling data allowed us to consider temporal patterns of promoter activity and individual TSSs in greater detail. The most frequent patterns for all promoters (peaked and broad) were 'all off' - that is, no utilization during any period (41%) - and 'all on' - that is, expression for the entire 24-hour duration of embryogenesis (5.8%; 272 TSSs). Patterns observed for more than five promoters are listed in Additional data file 6. In particular, we explored the profiles of genes with alternative promoters in greater depth (Additional data file 1). In this analysis, we excluded broad promoters from the set of 540 genes with alternative TSSs separated by at least 100 bp, on account of their lack of precise individual TSS resolution, and divided the remaining 407 genes into four categories. The first category consisted of 143 genes (35%) with no expression from any peaked promoters at any time point. The second category comprised 170 genes (42%) with exactly one alternative promoter active during embryogenesis. In this group, 75 genes showed expression at time point 1 and their promoters were thus maternally utilized. The third category included 20 genes (5%) with more than one but not all alternative peaked promoters utilized during embryogenesis. The remaining 74 genes (18%) in the fourth category had all alternative peaked promoters utilized at some time during embryogenesis.

**Figure 8:**
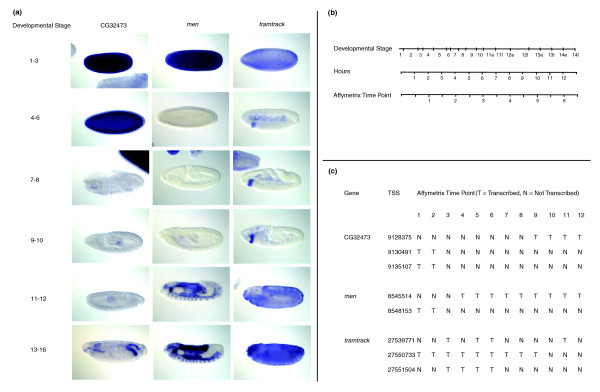
Differences in the temporal activity of alternative TSSs correspond to distinct patterns of gene expression. **(a) ***In situ *expression patterns of genes with alternative TSSs. *In situ *images showing the spatiotemporal expression of the CG32473, CG10120 (*men*), and CG1856 (*tramtrack*) genes during development [48]. **(b) **Correspondence between time period and developmental stage. As reference, the timing of developmental stages of the *Drosophila *embryo is matched to a timeline of 1-hour intervals and the Affymetrix 2-hour increment time course. **(c) **Utilization patterns as measured by the tiling array. The TSSs identified from the most frequent 5' EST ends are listed for each gene. The patterns of peaked promoter utilization detected on the tiling array are noted according to the 12 time points measured during embryonic development. Tiling array data showed that the peaked promoter of TSS#1 was utilized at time points 3, 5, 6 and 11 (hours 4 to 6, 8 to 12, and 20 to 22), TSS#2 at 1 to 9 (hours 0 to 18), and TSS#3 was used at time points 3 to 6 (hours 4 to 12). While the pattern of utilization of the promoter of TSS#1 flipped at time points 4 and 11, the patterns for both TSS#2 and TSS#3 were contiguous. TSS#2 is maternally inherited and the utilization of its promoter extends through early zygotic stages, while the utilization of the others starts after 4 hours and is active for a shorter time. Notably, the peaked promoter of TSS#2 was the only one without a (conserved) INR motif.

For the 74 genes in the fourth group, we examined the onset of utilization, as defined by the first time point in which utilization lasted at least 4 hours, or 2 periods. This removed isolated and thus potentially erroneous calls. There were 30 genes with the same onset time across alternative peaked promoters, albeit with different durations of utilization. The temporal utilization of the 44 genes with different onset across alternative peaked promoters was typically a combination of both maternal and zygotic utilization. For two candidate genes in particular, CG10120 (*men*), and CG32473, different peaked promoters corresponded to completely non-overlapping periods of activity. Available RNA *in situ *images [48] beautifully illustrated that the activity of distinct alternative promoters is associated with different spatiotemporal expression patterns (Figure 8). This switch in maternal versus zygotic promoter utilization mirrors the transcription of the well-studied gene *hunchback*, for which our dataset unfortunately did not contain enough ESTs to call TSSs. This analysis shows that dynamic properties of alternative promoter activity, such as onset and duration, are needed to properly characterize the regulation of transcription initiation during embryogenesis.

All three peaked promoters of the *ttk *gene were separated by at least 100 bp and each had an EST association with the embryo. Typical of the set of genes with the same EST conditions, temporal analysis of the alternative promoters revealed different patterns of utilization. Figure 8 shows the tiling array utilization and *in situ *staining of the complex patterns of gene expression observed for *ttk *during each stage of embryogenesis. While further experimental verification is needed to decipher the association between the spatiotemporal patterns and the utilization of each of *ttk*'s alternative promoters, RNA *in situ *images show the existence of distinct expression patterns at different stages that are consistent with the usage of alternative promoters [48].

#### Core promoters of maternally inherited and zygotically active transcription start sites have characteristic profiles of sequence elements

The presence of the two types of core promoters defined by different initiation patterns in *Drosophila *and vertebrates suggests that each may have a functional importance. To determine potential associations with specific conditions, we first compared the motif composition of 370 peaked promoters with head-specific TSS EST associations, and 765 peaked promoters with embryo-specific TSS EST associations (see Materials and methods). While we saw small differences between motif frequencies in the embryo and head-specific promoters, no clear trends for condition-enriched motifs were observed (Additional data file 1). This most likely resulted from the low resolution of these conditions, as both 'head' and 'embryo' encompass numerous tissues across various developmental stages.

**Figure 9:**
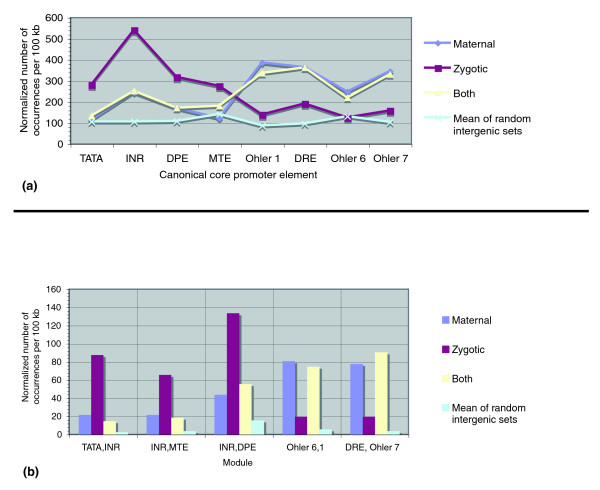
Elements in peaked promoters are associated with embryonic utilization. **(a) **Maternal and zygotic activity of peaked promoters corresponds to differences in element occurrences. The presence of eight sequence elements was evaluated in peaked core promoters of TSSs using PATSER. Core promoters were segregated into three groups based on their pattern of utilization (maternal, zygotic, both). Those showing no expression during the time course were excluded from this analysis. The normalized means of motif matches in three random intergenic sets are shown. **(b) **Regulatory modules also segregate by condition for peaked promoters. The numbers of occurrences of motif modules were evaluated in each of the three groups of peaked core promoters (maternal, zygotic, both) by counting the numbers of pairs of matches positioned in the designated order, with respect to the orientation of transcription.

We therefore examined the presence of sequence elements in the more precisely defined conditions that the tiling expression time course data allowed for, and analyzed 319 maternally inherited, 766 zygotically utilized, and 1,021 mixed maternally and zygotically active peaked promoters (see Materials and methods). We performed a concurrent analysis on 97 maternally inherited, 99 zygotically utilized, and 392 mixed broad promoters, to ensure that any identified associations of promoter elements with embryonic time points were consistent for different initiation patterns. The set of zygotically utilized peaked promoters showed a clear enrichment in the elements with strong positional bias - the TATA, INR, DPE, and MTE - and the maternally utilized sites had higher frequencies of the less location-biased elements (Ohler 1, DRE, Ohler 6, and Ohler 7; Figure 9a). While smaller differences in the frequencies of the elements were observed in the broad promoters overall, the same pattern of motif matches in the maternal versus zygotic conditions was found (data not shown). The association of the DRE, Ohler 6, and Ohler 7 motifs with maternal utilization was supported by a previous motif analysis that evaluated the significance of ImaGO terms in the *Drosophila in situ *hybridization database [49]. As this division in motif usage for maternal versus zygotic transcription was observed for both initiation patterns, it indicates that the repertoire of elements in the core promoters is determined by the different conditions. In χ^2 ^tests, the null hypothesis that initiation patterns and temporal conditions are independent of each other was rejected at (α = 0.05), indicating that maternal versus zygotic activity of core promoters and their initiation patterns are related to each other. For peaked and broad promoters with both zygotic and maternal activity, the frequencies of known elements agreed with those of the maternally utilized promoters (Additional data file 1). This relationship can be expected, as promoters with both patterns of utilization could in fact have resulted from the use of maternal promoters whose transcripts were not yet degraded within the cell. When compared to the numbers of occurrences in the random intergenic sets, the frequencies of the most common motifs were much higher overall in the promoters, although some of the less common motifs were in the range of frequencies observed for the random sites. This shows that when not in proper context, occurrences of the sequence elements are not as meaningful.

Akin to individual motif analysis, the occurrences of the TATA/INR, INR/MTE, INR/DPE, Ohler 6/1, and Ohler 7/DRE modules were evaluated separately for maternal and zygotic utilization (see Materials and methods). The results showed that the TATA/INR, INR/MTE, and INR/DPE modules had higher frequencies in the zygotically transcribed peaked promoters, and the Ohler 6/1 and Ohler 7/DRE modules had higher frequencies in the maternally utilized peaked promoters. Similarly, the numbers for promoters with both maternal and zygotic transcription initiation agreed with the maternally utilized module frequencies once again (Figure 9b). The same trends were observed for broad promoters (data not shown). In summary, these findings therefore complement the associations of initiation patterns with motifs, and propose that specific core promoter elements are more frequently utilized during different stages of development.

## Discussion

The identification of 5,665 TSSs from hierarchical 5' EST clustering provides a comprehensive map of reliable TSSs in *D. melanogaster *that should serve as a useful resource for information regarding condition-specific transcription initiation, and for computational modeling of promoter regions. Nevertheless, the saturation of the *D. melanogaster *transcriptome by available ESTs is certainly incomplete, and additional TSSs will certainly exist beyond the high quality set identified in this work. While promoters of alternative TSSs that are active under different spatiotemporal conditions have been reported for several individual fly genes [27,29], our analysis here establishes distinct spatiotemporal utilization of alternative promoters as a common feature in *D. melanogaster*. Some individual designations may result from biases due to the comparatively low coverage of ESTs or, in the case of the tiling arrays, from transcript expression levels falling below the sensitivity of the microarrays; however, the overall results strongly indicate that usage of many alternative promoters is condition-dependent. In humans, previous work has shown that the aberrant use of alternative promoters is associated with various diseases, such as cancer [26]. Genomic similarities that can be observed in the usage of promoters of alternative TSSs under different conditions in both humans and *Drosophila *may provide insight into downstream effects on transcripts and the mechanisms governing disease (Additional data file 1).

The promoters of alternative TSSs may also be utilized under the same broad EST-derived conditions. In fact, there was a higher than expected number of genes with alternative TSSs having the same condition associations. Alternative TSSs with the same condition associations may result from a series of point mutations, or be created anew through promoter sequence duplication [45]. In cases where there is no selective pressure to maintain all alternative sites, the promoters should exhibit a lower level of sequence conservation. This was in fact what we observed for the motifs of broad promoters in our set. On the other hand, there are cases in which the functional maintenance of several peaked promoters is linked to the same condition, and the promoters of these genes should exhibit a higher level of conservation. As an example, the enhancer for the *yellow *gene has been shown to interact with a promoter in *cis *and a promoter in *trans *based on core promoter composition [50]. A possible experimental validation of specific expression patterns linked to alternative promoters includes RNA *in situ *hybridization during different stages of fly embryogenesis [48,51]. *In situ *images are able to capture spatial gene expression patterns at a much higher resolution than ESTs and microarrays. Our study provides promising candidates for the design of isoform-specific probes, which would link differences in the spatial and temporal expression of transcripts of the same gene to different promoters.

*Drosophila *core promoters distinguish themselves from other eukaryotic species investigated so far by being defined by a repertoire of well-known sequence motifs. Recent work has shown that core transcriptional complexes are remodeled in specific cell types in both mammals and flies [34,52]. Here, we examined differences in motif frequencies and patterns of spatiotemporal utilization of peaked and broad promoters, which complements a concurrent recent study that explored how promoter motifs relate to one another across alternative promoters and adjacent genes [53]. We showed that peaked promoters have higher frequencies of the location-specific motifs (TATA, INR, DPE, MTE) and their corresponding modules, and higher levels of zygotic utilization. The importance of the location of elements in peaked promoters with respect to the TSS may reflect the binding architecture of zygotic-specific TAFs in RNA pol II. As broad core promoters surrounding maternally inherited sites have a higher number of occurrences of motifs with weaker positional bias (Ohler 1, DRE, Ohler 6, Ohler 7) and their modules, this suggests the hypothesis that larger regions of the DNA may be accessible at these locations. The localization of nucleosomes or specific chromatin marks may affect the accessibility of the DNA under specific conditions and locations, and explain the presence of specific initiation patterns [23,24]. In addition, a previous study suggested that the promoters recognized by TBP-related factor 2 up-regulate genes required for specific developmental pathways and may be involved in chromatin organization in mammalian gonads [25].

Our findings suggest that the core promoters of peaked TSSs in *Drosophila *are functionally equivalent to those of the single dominant peaked TSSs in vertebrates. The peaked promoters in both *D. melanogaster *and vertebrates have single, well-defined sites of initiation, contain location-specific motifs, and are associated with similar functional subsets of genes. Here, we showed that peaked *D. melanogaster *promoters are utilized zygotically, confirming previous findings that the promoters of genes with the INR and DPE are associated with developmental regulation and that the TATA is overrepresented in terminally differentiated tissues, such as the cuticle, and endocrine glands [19,21]. In vertebrates, peaked promoters are known to have an association to more tightly regulated transcripts [12]. In *Drosophila*, developmentally regulated genes were later shown to be associated with stalling of the RNA pol II machinery [22], and a circuit involving the TBP, Mot1, and NC2 that controls the regulation of DPE-dependent versus TATA-dependent transcription was shown to exist [54]. This suggests that a larger network regulates the transcriptional balance between functional classes of core promoters. As this analysis characterized individual sites of transcription initiation, and previous studies evaluated associations using whole genes in *Drosophila*, the functional associations of peaked promoters with developmental regulation and terminally differentiated tissues should be explored in greater depth. Our current set of peaked TSSs may change with additional data, as more detailed information on initiation events may lead to reassignments of patterns to promoters.

Similarly, we propose that the promoters of broad TSS cluster groups in *Drosophila *are functionally equivalent to broad regions of initiation in vertebrates [12]. Both of them are composed of multiple initiation sites, with no fixed spacing between them, contain motifs without a location enrichment, are void of the location-specific motifs, such as the TATA, and are present in similar functional subsets of genes. By showing that broad promoters are maternally utilized in *Drosophila*, this work supports previous studies showing that core promoter motifs without a location enrichment are utilized in the embryo, and are associated with housekeeping functions, such as DNA repair and translation, and the proteins necessary to perform them, such as the components of RNA pol II, and mitochondrial proteins [19,21]. Housekeeping genes with ubiquitous expression are associated with actively transcribing RNA pol II in *D. melanogaster *[22], and with broad patterns of initiation in vertebrates [12]. Furthermore, in our analysis, broad promoters were found to contain higher densities of the most frequent motifs and modules. As they define larger domains, broad promoters may be susceptible to higher probabilities of gaining motifs and modules. It will be interesting to explore whether, similar to other genomic properties, including gene family sizes [55] and protein folds [56], the relationship between motif density and genomic span of initiation is scale free.

It is important to recognize, however, that we are comparing the functional usage of each 'type' of core promoter across *Drosophila *and vertebrates, and not the actual sequence features that comprise them, as *Drosophila *and vertebrates have core promoter sequence features that are uniquely adapted to the transcription initiation machinery of each species. For instance, out of the eight motifs used in this study, only three motifs (TATA, INR, and DPE) have been shown to be functionally relevant for transcription initiation in vertebrates [19]. In turn, other sequence elements that play an important role in vertebrates, such as the downstream core element DCE, are absent in *D. melanogaster *[57]. The most salient difference between fruit fly and vertebrate promoters regards the presence of CpG islands. In vertebrates, CpG islands are characteristic of broad initiation regions, and are less frequent in peaked promoters, while in *D. melanogaster*, CpG islands do not exist, and peaked promoters have higher frequencies of G and C than those of broad promoters. This may indicate that the shape of promoters may be independent of the functional properties of CpG islands. The core promoter motifs may have been decoupled from CpG islands, or the properties of CpG methylation, selectively in the evolutionary history of *D. melanogaster*, as many other insect taxa have CpG methylation and orthologous proteins that catalyze it in vertebrates [58,59]. Furthermore, the core promoter motifs may be more dependent on the epigenetic features of the genome, such as the organization of histones and histone methylation, rather than on the properties of the DNA sequence itself.

Our study provided a high-quality data set to assess the conservation of core promoter elements across the recently published 12 *Drosophila *genomes. As we have experimental data for one species, we can only evaluate the loss of a *D. melanogaster *site in the corresponding location in another species. The fraction of candidates with non-conserved promoter elements in the *melanogaster *subgroup (approximately 10% depending on the motif and species) agrees with the turnover frequency measured by the ChIP-validated Zeste binding site [60]. The observed conservation levels drop drastically outside the *melanogaster *subgroup. A larger evolutionary effect in more distal species is certainly expected, but the recently observed low performance of multiple alignment algorithms on distal non-coding regions is likely to be a strong contributor to this observation [61,62]. Promoters of alternative TSSs, in particular those of broad TSS cluster groups, show a distinctly lower level of conservation of motifs across the 12 *Drosophila *genomes. This provides initial evidence of an average lower negative selective pressure on alternative and broad promoters, linked to the presence of functional motifs. A possible explanation for this effect was given in a recent TSS study on human and mouse, by using high-throughput CAGE sequence tags [63]. This study showed that alternative TSSs may arise in an intermediate stage of the process of TSS turnover. In support of this, an analysis of primate core promoters gave evidence for accelerated substitution rates [64].

The presence of canonical core promoter elements has shown that TSSs may be more dynamic than previously thought [65]. In addition to the effects discussed above, the promoters of alternative TSSs are involved in enhancer functionality [66,67], transcriptional interference [68], condition-restricted TAF utilization [69], and the maintenance of internal ribosome entry sites [70,71]. As the amount of data increases from capturing 4,000 genes in this study to the 13,767 genes present in the *D. melanogaster *genome, we expect the number of genes with alternative TSSs to scale accordingly. The first sets of 5' capped high-throughput transcript data have become available concurrently with our study, and such data will provide the necessary scale to follow up on our observations [72].

## Conclusions

Our study provides a genome-wide mapping of *Drosophila *TSSs and the distinct spatiotemporal conditions under which their promoters are utilized. Long underestimated in importance, differences in the motif composition of peaked and broad alternative core promoters have now been shown to be part of the complex spatiotemporal regulatory code of the eukaryotic transcriptome.

## Materials and methods

### EST filtering and clustering

We used EST alignments from *Drosophila *release 4.3 to identify TSSs, which enabled us to directly map our results to other available data sources (cross-species alignments and expression data). We filtered the ESTs in a four-step process by first eliminating ESTs that did not cover an intron splice junction. This reassured us that the remaining ESTs were produced from mature transcripts. Second, we removed ESTs having aligned fragments longer than 1,500 nucleotides, or a distance greater than 100 kb between any two fragments. This was done to exclude dubious ESTs that may incorrectly map to the genome. The parameter range of 50 to 100 kb corresponded to an upper bound of the genomic span of fly genes and was previously used as a natural cutoff for the determination of promoter co-regulation [40]. Third, we took out ESTs that aligned to multiple regions to ensure our set contained unambiguous locations. Fourth, we deleted ESTs with the most 5' location mapping to within 2 bp of the start of a downstream exon or transposon, as annotated in release 4.3. This served to eliminate incomplete ESTs, and those utilized by transposons. The 157,093 ESTs that remained were deemed highly confident in mapping to the most 5' ends of coding transcripts.

We implemented a hierarchical clustering strategy to define individual TSSs (Figure 2). We first parsed the ESTs by associating each of the 157,093 filtered ESTs with corresponding genes and dividing all of the ESTs for each gene into broad windows. Adjacent ESTs that were less than 100 bp apart were assigned to the same window, while adjacent ESTs greater than 100 bp apart were assigned to different windows. The window size of 100 nucleotides is a rule-of-thumb standard that has also been employed by EPD to specify broad regions of transcription initiation [8]. Moreover, the known sequence features directly involved in transcription initiation are all located within ± 50 nucleotides from the TSS, and the core promoter region of each TSS is generally defined to be approximately 100 bp in size. The genomic position of the 5' end of each EST alignment is referred to as the EST location.

We next computed the standard deviation of EST locations, and iteratively divided windows into smaller clusters until each had a standard deviation of less than 10. We refer to all of the clusters and sub-clusters having a standard deviation less than 10 by the term (sub-)cluster. This was done to discriminate regions of high localized EST frequency from broad regions with low EST frequency. It also served to separate singleton EST outliers into separate (sub-)clusters. The choice of 10 as standard deviation parameter corresponds to a variance of 100 bp and, thus, the size of a core promoter, as defined above.

### Transcription start site identification from EST clusters

We identified TSSs from the (sub-)clusters using four criteria. First, we found the location with the highest frequency of ESTs in each (sub-)cluster, and removed (sub-)clusters with a maximum frequency at a single site of less than 2. This criterion selected only those (sub-)clusters with consistently and reproducibly utilized TSSs. If two or more sites were tied for having the highest frequency of ESTs, the upstream site was chosen.

Second, to ensure that predicted locations coincided with the beginning of full-length transcripts, we selected sites that had to be supported by either at least three ESTs from a 5' capped library sequenced by RIKEN [5], or two RIKEN ESTs and a third EST within 5 bp from any non-RIKEN, non-capped library. For EST clusters without RIKEN ESTs, sites had to be supported by either three ESTs within five nucleotides of the 5' end of the cluster, or have at least half of the ESTs within a (sub-)cluster falling within five nucleotides of each other.

Third, if a cluster contained several TSSs identified for more than one (sub-)cluster, we placed a new window starting at one TSS and ending at the second TSS. If the standard deviation of this new window was less than the cutoff of 10, we kept the site with the higher frequency of ESTs as the TSS and removed the second location from the dataset. If the standard deviation of the new window was greater than 10, we kept both locations as TSS candidates. This eliminated closely spaced TSSs from adjacent (sub-)clusters.

Fourth, we required sites to be upstream of a start codon annotated for the gene in release 4.3. Because ESTs do not span the entire length of a transcript, we generally do not know what downstream isoforms correspond to the TSSs. For this reason, we conservatively required TSSs to be upstream of the most downstream start codon. If any of these criteria were not satisfied, we declared the (sub-)cluster to not have any conclusive TSSs and removed it from further analysis.

### Motif presence and conservation analysis

We applied the program PATSER [73] to the plus strand of the core promoter region [-60,+40] bp immediately surrounding the identified TSSs and the most 5' sites in Flybase, to look for hits to previously published position weight matrices above a threshold. For broad TSS cluster groups, promoter sequence [-60] bp of the most upstream TSS to [+40] bp of the most downstream TSS in the cluster group was extracted. To assess the strength of enrichment and conservation of motifs, we extracted 100-bp sets of sequences surrounding three randomly selected intergenic sets of sites, and repeated motif searches on these sets.

We used relative frequency matrices for eight core promoter motifs reported by Ohler *et al*. [18] and that were confirmed by analyses of other groups, for example, Fitzgerald [19]. We estimated set-specific mononucleotide backgrounds to account for varying AT content in the promoter sequences we analyzed (our TSS set; Flybase TSSs; and the random intergenic set). Score thresholds were individually chosen for each position weight matrix, always corresponding to a *P*-value of 10^-3 ^for the expected false positive hit per nucleotide. As seen in Figure 5b, motif matches in random intergenic regions agreed very well with the expected false positive rate. Motif matrices were taken from Ohler *et al*. [18], with one modification. The DPE as reported in that study is a composite of the closely spaced MTE and DPE elements (this can clearly be seen when comparing motif 9 (DPE) and motif 10 (MTE) with previous DPE consensus motifs), which is likely a side effect of the MEME motif-finding strategy employed in that study. To avoid confounding results by overlapping matches, we shortened both DPE and MTE to eight-nucleotide non-overlapping motifs. All frequency matrices and background models are provided in Additional data file 3.

Preferred motif positions were defined differently for location-specific and non-location-specific core motifs. For TATA, INR, DPE and MTE, we used the ten-nucleotide window with the highest number of motif matches in our *D. melanogaster *TSS set (-38 to -29 for the TATA box starting position, -4 to +6 for the INR motif, +14 to +23 for the MTE, and +21 to +30 for the DPE). These windows overlapped the most enriched motif locations as identified in the Flybase-defined promoter analysis of Fitzgerald *et al*. [19]. For the other four motifs, we used the 20-nucleotide windows as defined in that study (Ohler 1, -20 to -1; DRE, -60 to -41; Ohler 6, -60 to -41; and Ohler 7, +1 to +20). Note that we restricted motif matches to the preferred windows in some but not all analyses; in particular, preferred windows are somewhat less meaningful when dealing with broad cluster groups that do not exhibit a single initiation site.

For the conservation analysis, we first obtained orthologous regions across the other 11 species [14] using alignments computed by Multi-LAGAN [74]. Then, we selected promoters of TSSs having alignments in all 12 species, which led to a reduced set of 4,243 TSSs, with 2,075 genes with one TSS and 1,100 genes with more than one. As described above, we scanned orthologous regions in each species for motif hits above the threshold. For the location-specific motifs (TATA, INR, DPE, MTE), we identified matches in the *D. melanogaster *sequences within the 10-nucleotide preferred windows as defined above; for the other four motifs, we used the most-enriched 20-nucleotide windows [19]. Then, we assessed whether motif matches in *D. melanogaster *were located at corresponding positions in any of the other 11 genomes. Following the example of [60], we allowed for ± 5 nucleotides to account for possible small errors in the local alignments at the site of a motif match. In this way, we assessed whether a presumably functional motif, defined by the experimentally deduced location of the TSS and the occurrence of a motif match in the preferred position, was still detected in a second species, or potentially lost.

### Shannon entropy to measure condition enrichment

We assessed the condition association of TSSs by computing the Shannon entropy of the ESTs of each (sub-)cluster from which they were identified, using a protocol following previous methods [75]. First, we defined:



for (sub-)cluster *tss*, condition *i*, where *N*(*tss*,*i*) = the number of ESTs in each (sub-)cluster *tss *and condition *i*, *x*_*i *_= the number of ESTs for one condition across all (sub-)clusters, and 5,665 = the total number of (sub-)clusters in the analysis. In other words, *w*(*tss*,*i*) represents the normalized expression counts of the ESTs by condition and the overall size of the dataset. Next, we obtained the probability of observing an EST for each condition in a (sub-)cluster:



for *N*_*tss *_= the total number of ESTs in the (sub-)cluster across all conditions. To avoid arbitrarily low entropy values, we smoothed the data for conditions with no ESTs by setting *P *(*i | tss*) = 0.001. We calculated the entropy:



by summing across all conditions *i *for each *tss*. Then, we penalized entropy values to account for the disparity in sampling depth across conditions:



Lastly, we characterized the condition utilization of each (sub-)cluster by using an EST frequency threshold and the penalized entropy values, *Q*_*i*,*tss*_. Only (sub-)clusters having at least three ESTs from a condition were evaluated further to prevent potential false assignments due to a low frequency of ESTs. The entropy values for *H*_*tss *_ranged from 0 to log_2_(c), for *c *= the number of conditions. In our analysis, *c *= 9 (eight distinct conditions and one diverse condition), and values for *Q*_*i*,*tss *_ranged from 0 to log2(9) - log2(0.0001), or 16.458.

*Q *values naturally segregated into three clearly distinct groups (Figure S4 in Additional data file 1). Entropy values close to zero signified (sub-)clusters with ESTs mainly from one condition. Larger entropy values characterized (sub-)clusters with ESTs that were more broadly distributed across libraries, but still mainly concentrated in one or two conditions. The greatest entropies denoted (sub-)clusters with ESTs spread across many of the eight conditions. On account of these groups, we classified the TSS associations into three categories (condition-specific, condition-supported, and mixed) based on chosen cutoffs of *Q*_*i*,*tss*_. TSSs were declared condition-specific if 0 ≤ *Q*_*i*,*tss *_≤ 1, and there were less than two ESTs from other conditions, and condition-supported if 0 ≤ *Q*_*i*,*tss *_≤ 1, and more than two ESTs were generated from other conditions. We also classified TSSs as condition-supported if 1 ≤ *Q*_*i*,*tss *_< 10. TSSs with *Q*_*i*,*tss *_≥ 10, and those that were classified as specific or supported by more than two of the eight distinct conditions, were deemed to have mixed association. Finally, TSSs that were specific or supported by the diverse condition were assigned mixed association by default.

### Evaluating temporal usage of promoters by Affymetrix tiling arrays

Our analysis is based on a published embryonic time course, and we evaluated promoter activity by using reported normalized intensity values of 25-bp long probes [40]. The spatiotemporal utilization of the most upstream TSS in a broad TSS cluster group was chosen to characterize the whole group, as the low resolution of the Affymetrix tiles did not permit an evaluation of individual closely spaced TSSs. This resulted in 4,664 well-separated promoters. For each promoter, the median of fluorescence intensity of three downstream tiles of the TSS was subtracted from the median of fluorescence intensity of three upstream tiles from the TSS, with respect to the orientation of transcription. Tiles containing the TSS location were excluded from the analysis because we did not expect such probes to show consistent expression.

Due to the differing levels of total transcription across the 12 2-hour periods, cutoffs were determined independently for each time point. A mixture model of two Gaussians was fit to the differences of each time point using expectation maximization. The point of intersection of the two Gaussians was rounded up to the nearest.5 and declared the threshold (Additional data file 1). All promoters having differences greater than the threshold were deemed transcribed (T) for that time point. Promoters having differences in median fluorescence intensity less than the time point-specific threshold were declared non-transcribed (N). To determine the expected fraction of false predictions at these cutoffs, we randomly selected 4,664 random intergenic sites as a control dataset. For each of these sites, we evaluated the difference in fluorescence intensity by using the same methodology and threshold values, and assuming the sites had positive orientation.

The fraction of promoters transcribed at each time point was determined by dividing the number of transcribed promoters at each 2-hour period by the total number of promoters. A paired *t*-test was applied to the fractions of transcribed peaked versus broad promoters to evaluate statistical significance. The same strategy was used to compare the fraction of peaked versus broad promoters with embryo EST associations over all 12 time points, and to compare the total number of initiation sites with embryo EST associations to those without. For the evaluation of the association of both types of promoters with embryo and non-embryo ESTs associations, without the tiling array data, a χ^2 ^test with Yates' continuity correction was applied. A Bonferroni correction was used in all tests, reducing the effective significance level to 0.01.

In the core promoter analysis, maternally inherited sites were defined as having utilization during time points 1 and/or 2 in the tiling array. Sites with zygotic transcription were required to have utilization during at least one 2-hour period from time points 4 through 12, and sites with both maternal and zygotic utilization needed to satisfy both requirements. The promoter element matches previously identified were summed up separately for these three sets. As the initiation pattern does not play a role with regard to random intergenic sites, the mean numbers of elements identified in the 1,299 random sites served as a baseline. To test the relationship between initiation pattern and condition, we summed the normalized frequencies of the location-specific motifs (TATA, INR, DPE, and MTE) and non-location bias motifs (Ohler 1, DRE, Ohler 6, Ohler 7) in peaked promoters with maternal (respectively zygotic) utilization, and in broad promoters with maternal (respectively zygotic) utilization, and performed a χ^2 ^test on both 2 × 2 contingency tables.

## Abbreviations

BDGC: Berkeley *Drosophila *Genome Collection; CAGE: capped analysis of gene expression; ChIP: chromatin immunoprecipitation; DPE: downstream core promoter element; DRE: DNA replication element; EPD: Eukaryotic Promoter Database; EST: expressed sequence tag; GO: Gene Ontology; INR: initiator; MTE: motif ten element; RNA pol II: RNA polymerase II; TAF: TBP-associated factor; TBP: TATA-box binding protein; TF: transcription factor; TRF2: TBP-related factor 2; TSS: transcription start site; *ttk*: *tramtrack*.

## Authors' contributions

UO, PT and EAR conceived, designed, and coordinated the study. EAR clustered the ESTs, identified the TSSs, assigned EST condition associations, evaluated promoter utilization from the tiling arrays, and compared the presence of motifs in promoters with different patterns of initiation and spatiotemporal utilization. UO and HY evaluated the conservation of motifs across species. WHM performed the GO analysis. EAR and UO wrote the manuscript.

## Additional data files

The following additional data are available with the online version of this paper: Tables S1 to S4 and Figures S1 to S4, including detailed information on the comparison of the identified TSS locations to other genomic promoter resources and on the condition-specific activity of TSSs as determined by ESTs and tiling arrays (Additional data file 1); a list of the initial groupings of ESTs, the (sub-)clusters created after clustering, and the TSSs chosen from each (sub-)cluster (Additional data file 2); a list of the position weight matrices and the background models used in PATSER to search for motifs in the core promoters of the most 5' sites in Flybase, the identified TSSs, and the random intergenic sites (Additional data file 3); a list of the gene, chromosome, orientation, and condition association as determined by Shannon entropy for each individual TSS (Additional data file 4); a list of the gene, chromosome, orientation, and temporal pattern of utilization determined by the tiling arrays for peaked and broad promoters (Additional data file 5); a list of the patterns of utilization across the 12 development periods that occur at least 5 times in the set of peaked and broad promoters (Additional data file 6).

## Supplementary Material

Additional data file 1 Table S1: false positive estimates of TSS assignments by condition. To assess the validity of the TSS condition assignments, we performed 100 random permutations of condition labels from the (sub-)clusters and evaluated their associations using the same methodology as for the identified TSSs. The numbers of false positives (column 3) were empirically estimated as the mean number of sites having a specific association with each condition (column 1) across all 100 random permutations. The false positive rate (column 4) was calculated by dividing the number of false positives by the number of identified TSSs that were observed to have the condition association (column 2). Table S2: GO enrichments for genes with different condition associations for alternative TSSs. The table lists all significant GO categories for genes with alternative TSSs associated with specific conditions, at a false discovery rate cutoff of 0.1, and present in more than five genes. Table S3: embryo associations confirm utilization patterns of known genes. We compared the embryonic utilization patterns previously observed for known genes to those identified using EST and Affymetrix tiling array data. Analysis of genes with at least one TSS having an EST embryo association (column 3), and promoter utilization in at least one tiling array time period (column 4) agree with previously reported expression patterns from *in situ *images (column 5) [48], and published reports (column 6). Table S4: false positive approximations of embryonic temporal promoter assignments. We evaluated the expected number of false positive temporal expression assignments for the set of promoters of 4,664 identified TSSs across 12 developmental periods (column 1) corresponding to 2-hour increments during embryogenesis (column 2). We chose 4,664 random intergenic sites and found the difference in median fluorescence intensities of neighboring tiles for each of the 12 time points. The differences in fluorescence intensities were compared to the difference thresholds (column 3) used to classify the set of 4,664 promoters. Random intergenic sites with fluorescence intensity differences above the threshold were counted as false positives. For each time point, the total number of false positives (column 4) was divided by the total number of random intergenic sites to approximate the rate of false positives (column 5). Figure S1: alternative TSSs and alternative promoters are widely distributed across the genome. For each chromosome, the number of genes with one TSS location (blue) and more than one (that is, alternative) TSS location (red) were counted. Genes having alternative TSSs were divided into two groups according to the number and type of promoters: those having one broad promoter (yellow) and those having alternative promoters of the peaked or broad type, or any combination thereof (green). With the exception of chromosome 4, the overall fraction of genes with alternative TSSs ranged from 28 to 32%, and the fraction of genes with alternative promoters was 12 to 14%. Chromosome 4 is much smaller in size than the other *Drosophila *chromosomes, and had an elevated percentage of genes with alternative TSSs (19 out of 38; 50%) and alternative promoters (34%), possibly due to the small sample size. Figure S2: evaluation of TSS quality. The quality of the TSS calls was evaluated by comparing the locations of initiation sites across databases and the frequencies of elements in the core promoter sequences surrounding them. **(a) **EPD location differences. Each of the 1,840 EPD TSSs was compared to the set of identified TSSs that were on the same chromosome. The difference in location of the closest identified TSS was taken from each EPD TSS, with the identified TSS as reference position (0). Differences ranged from 0 to greater than 1,000 bp. The plot covers a region of ± 20 nucleotides, which covers 76% (1,404) of EPD start sites. **(b) **Flybase location differences. All TSSs in Flybase that were upstream of the most downstream start codon, and did not map to a start codon location, were selected for comparison. Each of the TSSs identified by the hierarchical clustering strategy was compared to all of the Flybase TSSs listed for the same gene. The smallest difference in location between the Flybase TSS and the selected TSS was calculated at 1-bp resolution using the selected TSS as a reference point (0). The orientation of transcription of each gene was used to determine the orientation of the differences. A negative difference corresponded to a Flybase TSS being located upstream of the selected TSS, and a positive value signified that the Flybase TSS was downstream of the selected TSS. The plot covers a region of ± 300 nucleotides, which covered 79% (4,406) of TSSs matching to Flybase start sites. Compared to EPD, differences in start site locations are thus one order of magnitude larger at roughly the same coverage. **(c) **Presence of core promoter elements. For 2,725 genes with exactly one TSS in our set and an annotated initiation site in Flybase, motif matches were identified in the preferred windows in their core promoter sequences using separate zero order Markov models as background. There is a consistently higher number of motif matches in the promoters of the TSSs identified here, compared to those of the TSSs from the Flybase 5' end annotations. Figure S3: sequence elements in preferred windows of peaked promoters preserve trends of motif associations. **(a) **Associations of element occurrences. Motif matches were constrained to their preferred windows in peaked core promoters and normalized to the number of occurrences per 100 kb (see Materials and methods). The mean number of occurrences across the three random intergenic sets is shown. **(b) **Correspondence of elements to embryonic utilization. The set of peaked core promoters was divided into three groups according to the their pattern of embryonic utilization (maternal, zygotic, or both). The numbers of elements in the preferred windows of each group are shown. Figure S4: Shannon entropy values segregate into three groups. The distributions of ESTs in the (sub-)clusters used to call TSSs were evaluated using Shannon entropy. As an example, the figure shows the entropy histogram for the embryonic condition with bins of size 0.5. The *Q*_*Embryo*,*tss *_values naturally separate into three groups: those less than 1, those between 1 and 10, and those greater than 10. The large frequency of *Q*_*Embryo*,*tss *_values between 13 and 13.5 is an artifact resulting from using 0.0001 to smooth *P*(*i *| *tss*) for (sub-)clusters containing ESTs mainly from one non-embryo library. Click here for file

Additional data file 2 Genomic locations and the frequencies of ESTs from each library are given for the initial groupings of ESTs, the (sub-)clusters created after clustering, and the TSSs chosen from each (sub-)cluster. Click here for file

Additional data file 3 All motif matches in the peaked and broad promoters are included, regardless of preferred windows. Promoters without at least one motif match are excluded from the file. Click here for file

Additional data file 4 Gene, chromosome, orientation, and condition association as determined by Shannon entropy for each individual TSS. Click here for file

Additional data file 5 Gene, chromosome, orientation, and temporal pattern of utilization determined by the tiling arrays for peaked and broad promoters. Click here for file

Additional data file 6 Patterns of utilization across the 12 development periods that occur at least 5 times in the set of peaked and broad promoters. Click here for file
