# Supplementary material for: Motif composition, conservation and condition-specificity of single and alternative transcription start sites in the Drosophila genome
Source: Genome Biol. 2009 Jul 9;10(7):R73. doi: 10.1186/gb-2009-10-7-r73 (PMC2728527; doi:10.1186/gb-2009-10-7-r73)
Supplement: Additional data file 1 — Table S1: false positive estimates of TSS assignments by condition. To assess the validity of the TSS condition assignments, we performed 100 random permutations of condition labels from the (sub-)clusters and evaluated their associations using the same methodology as for the identified TSSs. The numbers of false positives (column 3) were empirically estimated as the mean number of sites having a specific association with each condition (column 1) across all 100 random permutations. The false positive rate (column 4) was calculated by dividing the number of false positives by the number of identified TSSs that were observed to have the condition association (column 2). Table S2: GO enrichments for genes with different condition associations for alternative TSSs. The table lists all significant GO categories for genes with alternative TSSs associated with specific conditions, at a false discovery rate cutoff of 0.1, and present in more than five genes. Table S3: embryo associations confirm utilization patterns of known genes. We compared the embryonic utilization patterns previously observed for known genes to those identified using EST and Affymetrix tiling array data. Analysis of genes with at least one TSS having an EST embryo association (column 3), and promoter utilization in at least one tiling array time period (column 4) agree with previously reported expression patterns from in situ images (column 5) [48], and published reports (column 6). Table S4: false positive approximations of embryonic temporal promoter assignments. We evaluated the expected number of false positive temporal expression assignments for the set of promoters of 4,664 identified TSSs across 12 developmental periods (column 1) corresponding to 2-hour increments during embryogenesis (column 2). We chose 4,664 random intergenic sites and found the difference in median fluorescence intensities of neighboring tiles for each of the 12 time points. The differences in fluorescence intensities were [file gb-2009-10-7-r73-S1.doc]

### *Additional data file 1: Supplementary Methods and Figures*

### Data

Our results are based on the dataset of *D. melanogaster* ESTs in the Berkeley *Drosophila* Genome Collection [16, 39]. A large subset of EST libraries was obtained with a protocol to capture capped full-length transcripts. Libraries were deposited by various research teams and cover a broad spectrum of different conditions (Figure 1). The Oliver Lab generated ESTs from the testes library BS, which is of interest because germline tissues exhibit a sex-specific transcriptional program [52, 69]. Exelixis generated two immune-response related libraries, EN and EC, from LPS induced mbn2 cells, and fat bodies of third instar larva challenged with gram +/- bacteria, respectively. There were 11 smaller libraries of ESTs grouped together into the library “OTHERS” and assigned the default condition diverse. ESTs from the BQ and EK libraries were also labeled with the diverse condition because they were derived from unknown and multiple sources (*Drosophila* embryos, imaginal discs, and adult heads). Some library conditions corresponded to developmental stages of the fruit fly life cycle (embryo and the larva/pupa). Schneider cells were captured by immortalized cell lines obtained from late embryonic stages (20-24 hrs). The remaining five specific conditions corresponded to body parts of the adult fruit fly. More information about the EST libraries can be found at the BDGC [76].

**TSS Identification**

By designating TSS positions at the location of the highest EST frequencies within a clearly delineated cluster, instead of at every mapped location [5] or the most 5’ one [77], we were able to gain new insights on the architecture of core promoters and their associations to conditions. The two most sensitive parameters in the clustering algorithm were the standard deviation and minimum frequency. We selected informed values based on previous analyses of *Drosophila* core promoters, however, increasing or decreasing these values changes the number of TSSs identified. Given the amount of available data and correspondingly chosen clustering parameters, all TSS positions are separated by at least 20bp, with the consequence that motif assignments, which were restricted to small, preferred windows in the core promoters relative to the TSSs, could be made to individual sites. In mammalian studies on large-scale 5’capped transcript datasets, initiation sites were observed at many closely spaced locations and called at single-nucleotide resolution [13]. While some of the initiation frequencies at this resolution have been shown to be condition-specific, broader TSS initiation patterns may potentially be a result of some degree of sloppiness in the transcriptional machinery, and functional consequences of such differences on transcription are of yet unclear. With the data used in this study, we could not resolve whether start sites in flies are overall defined in tighter patterns, even if they are closely spaced to each other, or if additional data would lead to broader patterns.

### Comparison of Identified TSS Locations to Other Genomic Data Sources

The first source we considered was the Eukaryotic Promoter Database EPD [8], which has long been the reference source for computational sequence analyses and modeling the proximal core regions of promoters. The TSSs listed in EPD are the result of a strategy paralleling ours, using the oligo-capped EST subset from the BDGC. The promoters of TSSs in EPD are divided into three groups: those surrounding single transcription initiation sites, those around initiation regions, and those encompassing multiple initiation sites [8]. These categories correspond to peaked promoters, broad promoters, and alternative promoters in our set, and to single peak (SP), broad (BR), and more than one SP or BR in the vertebrate set [12]. EPD contains one-third the number of sites in our set, 1,926 TSSs, of which very few are alternative sites. To compare the locations of the identified TSSs to those in the Eukaryotic Promoter Database EPD [78], we downloaded 201 bp surrounding each TSS for 1,922 *D. melanogaster* promoters and aligned them to Release 4 with BLAST [79]. This was done to map entries to a common reference point because EPD does not use Flybase IDs for TSS identification. We removed 82 sequences with non-unique matches or less than 75% sequence similarity from the analysis, and compared the locations of the remaining 1,840 EPD entries to the closest TSSs in our set.

The distribution of distances between the locations of TSSs in our set and those in EPD showed a high percentage of overlap (Figure S2A), with the mode difference being 0bp. The mean location of EPD TSSs was ~35bp downstream of our identified TSSs. There were 577 EPD TSSs (31%) that mapped exactly to the identified TSS locations and 1,404 EPD TSSs (76%) within +/- 20bp. The promoters of the set of 1,404 TSSs were distributed across all three EPD classifications in the following way: 35% were single initiation sites, 26% were multiple initiation sites, and 39% were initiation regions. There were 136 EPD TSSs that were located within 21bp to 1,000bp from the closest TSS, and 300 EPD TSSs were farther than 1,000bp away. Such large distances are likely the result from comparing the closest TSS locations between our set and EPD, regardless of different gene associations. Instead of disagreements in calling the location of a TSS, this fraction rather indicates the number of distinct TSSs that are included by either us or EPD alone, but not both. TSSs in EPD were identified with the program MADAP [78], which requires a user-specified initial range of the number of TSS clusters, which is typically not known *a priori*. In contrast, the hierarchical clustering strategy that we used heuristically identifies TSSs using parameters based on genomic properties of core promoters, without any initial knowledge of the number of TSSs. Also, MADAP uses Gaussians to model EST distributions, regardless of fit, which may lead to calls of TSSs at the mean location instead of the most frequent one. In particular, isolated ESTs far away from a cluster have a large impact on the selection of the mixture model and may bias TSS locations, and may contribute to the differences observed between EPD and our set.

The second source to which we compared our set of TSSs was Flybase. Flybase is the most complete database of *Drosophila* transcript information to date. Rather than TSS annotations *per se*, Flybase contains the 5’ ends of genes or transcripts which have been annotated using a variety of experimental and computational methodologies, followed by manual curation [36]. To provide a balanced comparison of Flybase TSSs to identified TSSs, the 5’UTR files of the same release (4.3) were chosen for analysis. Flybase TSSs were extracted for each chromosome, and only those upstream of the gene’s most downstream start codon, but not mapping to a start codon, were selected for comparison. These criteria were in agreement with the standards used to identify the hierarchically clustered TSSs, and ensured the exclusion of obviously suspicious or incomplete transcripts. A set of 18,767 TSSs for 9,655 genes from Flybase remained for comparison, over three times the number of TSS candidates in our set. Because we mapped TSSs to Flybase gene IDs, we were able to compare 5,610 TSSs for 3,945 genes in our set, ~99% of the TSSs identified by hierarchical clustering, to comparable sites of the same genes in Flybase.

Akin to EPD, Flybase TSS locations showed a high percentage of overlap to the identified TSS locations (Figure S2B), with 720 Flybase TSSs mapping perfectly to identified TSS locations; 30% located within 20bp; and 54% less than 100bp from identified TSSs. This resulted in the mean location of Flybase TSSs 98bp upstream of identified TSSs. Only 148 Flybase TSSs (2.6%) were more than 1,000bp from the identified TSSs. Of the 148 sites, only 13 Flybase TSSs were upstream of ours, and the remaining 135 Flybase TSSs were located more than 1,000bp downstream of identified TSSs. They may be genuine alternative downstream TSSs with too little support to be included in our set, or incomplete transcripts. The differences in TSS locations in Flybase are likely to stem from the application of various criteria to annotate transcripts and their 5’ ends, rather than the application of one consistent method for TSS calling.

The mean location of Flybase 5’ ends falling ~100 nt upstream of ours most likely reflects the long-standing strategy to call the most 5’ location of any single piece of evidence as the 5’ end of the whole transcript, even if many ESTs align to a more downstream location. While this strategy certainly leads to correct calls in terms of the genomic span of a gene, it does not correspond to high-quality TSS calls. We recognize that experimental bias from PCR amplification may exist in the set of the most highly utilized TSSs [80], however, we believe it to be minimal, as the majority of differences between our TSSs and those in EPD and Flybase is less than 100bp. Such small differences in transcript size do not have a big impact on the rate of PCR amplification. As such, many analyses of CAGE data [13] now commonly define the most frequent tag location as TSS.

To further demonstrate the reliability of the identified TSSs, we examined the presence of reported core promoter elements. For the Flybase motif comparison, we determined the frequency of the TATA, INR, DPE, and MTE motifs that are known to occur at a narrow distance from the TSS, in their preferred 10nt windows in the promoters. In addition, we analyzed the occurrence of the Ohler 1, DRE, Ohler 6, and Ohler 7 motifs in their 20bp preferred windows as defined in [19] (see Additional data file 3), as they are known to occur more widely throughout the core promoter. To allow for a clear comparison, we contrasted the number of motif matches in core promoters of genes with single promoters in our set to the number of matches in the core promoters of the most 5’ TSS coordinates defined for the same genes in Flybase.

Figure S2C shows that each of the four location-specific canonical elements was ~1.6-2.4 times more frequent in the core promoters of most highly utilized TSSs, than in those surrounding the most 5’ sites in Flybase. For the more broadly occurring Ohler 1 and DRE, a similar trend was observed with 1.8 and 1.5 times more occurrences of motif matches in the core promoters of the most highly utilized sites, respectively. For Ohler 6 and Ohler 7, the differences in motif frequencies were not distinctive; however, these two motifs have little location-specificity (Figure 5), and the small differences in occurrences most likely reflect the breadth of locations of motif instances. The pattern that Flybase-derived promoters show a significantly lower number of known regulatory motifs has also been reported elsewhere [81]. Due to the stringent window sizes, motif frequencies were overall lower than in some previous estimates; for instance, following the criteria in [18], we detected an INR in 25% and a TATA box in 16.5% of promoters of genes with one TSS, which compares well with the presence of an INR in 26.3% and a TATA box in 19.3% of *melanogaster* promoters in the earlier study.

### TSS Condition Associations Derived From EST Libraries

We used Shannon entropy to evaluate the specificity of association of a TSS in one condition relative to the other conditions (see Materials and Methods). Entropy measures have been used to characterize the associations of genes to tissues in the mouse and human genomes using ESTs previously [75]. By assessing all TSSs, regardless of initiation pattern, we allowed for several alternative TSSs of the same gene to have different condition associations. For each condition, the penalized entropy values (Q_tss_) naturally segregated into three groups that we classified as: condition-specific, condition-supported, or mixed (see Materials and Methods, Figure S4). This classification scheme provides an initial framework for characterizing TSS condition associations, and ultimately promoter utilization. It can be easily expanded to future data sets and applied to other types of count data. Current TSS condition associations were made given the available set of ESTs, and may change with the inclusion of additional 5’ capped data.

The two main sources of library bias that can affect the determination of the

condition-specificity of the TSSs by applying Shannon entropy to the ESTs are library normalization and library size. While we recognize that a slight normalization bias may remain in each of the individual libraries, we do not believe that the condition-specificity assignments were significantly impacted by these different biases because multiple independent libraries were used to generate the collections of ESTs for each of the eight conditions. It is highly unlikely that the exact same normalization bias would be present in all of the independent libraries. The second source of bias that may affect TSS condition-specificity arises from using libraries of different sizes. The repertoire of ESTs used in this work is the largest amount of publicly available transcript data for *Drosophila* to date, but certainly does not saturate the transcriptome. As a consequence, differences in the numbers of ESTs from each cDNA library may affect the condition associations made using Shannon entropy. In an effort to minimize this bias, we removed alternative TSSs with very few ESTs because they had a higher probability of being affected by library size bias. We also penalized entropy values according to the sampling depth of ESTs in their corresponding libraries. In spite of these efforts, alternative TSSs may have condition associations simply due to the low resolution of the available data. For instance, alternative TSSs may have embryo-specific associations using the EST library information, but be utilized at different temporal stages of embryogenesis. We confirmed this phenomenon to some extent for the group of TSSs active during embryogenesis by using available expression data from whole-genome tiling arrays. However, higher resolution of data is needed across all body parts and time periods to gain deeper insight into the precise spatiotemporal utilization of TSSs.

We validated the significance of the TSS associations by performing 100 random permutations on condition labels to (sub-) clusters. For each permutation, we preserved the same partitioning of EST frequencies across the (sub-) clusters as in the identified set, and applied Shannon entropy to the (sub-) clusters to identify specific, supported, and mixed condition associations. We then fitted the total number of condition-specific associations across the 100 permutations to a Gaussian distribution and used the Gaussian to obtain an empirical P value. The number of 1,997 condition-specific TSSs that we identified was significantly higher than random permutations (p << .001) (Figure 6B). The mean number of condition-specific sites in the set of 100 random permutations was 215, leading to a false positive estimate of 10.8%. In turn, there were significantly fewer sites with mixed associations and condition-supported TSSs (p << .001 for both groups). We empirically estimated the number and rate of false positives for each condition individually (Table S1). These results indicate that any bias resulting from differences in library sizes is minimal, even for the smallest libraries. The numbers of condition-specific TSSs identified greatly deviate from expectation, and are thus, likely to reflect true patterns of transcription initiation.

| TSS Association | # Identified TSSs | Estimated # False Pos. | False Pos. Rate |
| --- | --- | --- | --- |
| Embryo | 1134 | 152 | 13.40% |
| Larva Pupa | 27 | 0 | 0% |
| Head | 522 | 63 | 12.10% |
| Ovary | 9 | 0 | 0% |
| Testes | 247 | 0 | 0% |
| Schneider Cells | 36 | 1 | 2.80% |
| Mbn2 Cells | 12 | 0 | 0% |
| Fat Body | 10 | 0 | 0% |

Table S1

To evaluate the statistical significance of the gene associations, we performed 100 random permutations of the pattern associations called in the identified set of 5,665 TSSs. Gaussians fit to the data had on average 242 genes with the same condition associations across alternative TSSs and 983 genes with different associations across alternative TSSs. The counts we observed in the real data significantly differed from these numbers (p << .001), and this was also the case when we repeated the analysis on permuted labels. This implies that alternative TSSs of the same gene have a higher than expected probability of being utilized in the same condition. However, as conditions defined by EST libraries are very coarse, this trend may not be observed with more precise conditions.

We also performed a Gene Ontology (GO) analysis to assess whether genes with alternative TSSs had enrichments for specific functional categories based on their distinct conditions [82]. GO assessments were made for each of the eight distinct conditions [82], and whole genes with more than one TSS or TSS cluster group were designated as having alternative TSSs. The foreground set consisted of genes with alternative TSSs associated with a specific condition. The background set included genes with associations to that condition plus all genes with a mixed condition association. We evaluated enriched terms on levels 2 and 3 of the GO hierarchy. The overrepresentation of terms in the foreground set when compared to the background set was measured using a hypergeometric test. To control for multiple comparisons, we applied a false discovery rate (FDR) threshold of 10% [83]. We also excluded terms with less than or equal to five expected occurrences. This showed enriched terms broadly reflecting functions required for the proper development of the embryo, suggesting that genes with alternative TSSs are significantly active during embryo-specific processes (Table S2).

| Condition | P value | FDR | No. Observed | No. Expected | GO Term |
| --- | --- | --- | --- | --- | --- |
| Embryo |  |  |  |  |  |
| Biological Process: |  |  |  |  |  |
| Level 2: |  |  |  |  |  |
|  | 0.000221 | 0.002040 | 35 | 19 | system process |
|  | 0.000572 | 0.004081 | 26 | 14 | behavior |
|  | 0.002412 | 0.006122 | 54 | 37 | cellular devel. process |
|  | 0.002576 | 0.008163 | 24 | 14 | cell motility |
| Molecular Function: |  |  |  |  |  |
| Level 3: |  |  |  |  |  |
|  | 4.29E+06 | 0.002380 | 29 | 12 | cytoskel. protein binding |
| Cell. Component: |  |  |  |  |  |
| Level 2: |  |  |  |  |  |
|  | 0.000626 | 0.004761 | 60 | 40 | membrane of cell |
| Level 3: |  |  |  |  |  |
|  | 0.000217 | 0.001923 | 27 | 14 | plasma membrane |
|  | 0.000386 | 0.003846 | 22 | 11 | plasma membrane part |
|  |  |  |  |  |  |
| Testes |  |  |  |  |  |
| Cell. Component: |  |  |  |  |  |
| Level 3: |  |  |  |  |  |
|  | 0.000241 | 0.003448 | 16 | 8 | cytoplasm |

Table S2

As discussed in the Background, a number of well-known genes involved in development are known to have developmentally regulated alternative TSSs associations. In *Drosphila*, there have been overall few well-studied examples of genes having differentially utilized promoters, compared to human and mouse [84]. This is especially true for the differential regulation of TSSs during embryogenesis, as the transition to zygotic transcription is known to cause the degradation of maternal mRNAs [85, 86]. While some of the well-known genes have too few ESTs to call their TSSs, we collected a list of 10 genes with known utilization during embryonic development, as verified by *in situ* and published sources (Table S3). The patterns of embryonic utilization for these genes in our dataset confirm previous observations and further suggest that the promoters of TSSs for these genes are developmentally regulated.

| Gene ID | Name | EST | Array | BDGP in situ | Developmental Regulation |
| --- | --- | --- | --- | --- | --- |
| CG10334 | spi | X | X | X | Rutledge et al, Genes Dev 1992 |
| CG1856 | ttk | X | X | X | Read et al, Mech Dev, 1992 |
| CG2671 | l(2)gl | X | X | Klaembt et al, EMBO, 1986 | Mechler et al, EMBO, 1985 |
| CG31243 | cpo | X | X | X | Bellen et al, Genes Dev, 1992 |
| CG3725 | Ca-P60A | X | X | X | Varadi A et al, FEBS Lett, 1989 |
| CG4898 | Tm1 | X | X | Hales et al, Dev Biol, 1994 | Hales et al, Dev Biol, 1994 |
| CG8989 | His3.3B | X | X | Feng et al, Genome, 2005 | Akhmanova et al, Genome, 1995 |
| CG9075 | eIF-4a | - | X | Hernandez et al, Proteomics, 2004 | Dorn et al, Mol Gen Genet, 1993 |
| CG9261 | nrv2 | X | X | X | Xu et al, Gene 1999 |
| CG9553 | chic | X | X | Cooley et al, Cell, 1992 | Cooley et al, Cell, 1992 |

Table S3

Associations were also confirmed for TSS associations to the other conditions. For the gene *Calmodulin* (CG8472), we identified two TSSs separated by 21bp. The upstream TSS had a mixed association, while the downstream promoter had LPS induced mbn2 support. Utilization of the downstream promoter agrees with the known activation of *Calmodulin* as part of the LPS induced mbn2 immune response [87]. For the gene CG11151, we also identified two TSSs separated by 100bp; the upstream TSS had a mixed association, and the downstream TSS was supported by the larval fat body, confirming reported expression of the gene in the lipid subproteome [88]. Condition associations may also provide new information about genes with limited or no molecular, biological, or cellular functional evidence in Flybase. As examples, the gene CG10510 had one TSS with a specific association to the testes, and the TSS for CG1814 showed support for the head condition.

### Temporal Utilization of Alternative Promoters During Embryogenesis

To examine initiation events at higher resolution, we used available Affymetrix whole-genome tiling arrays of *D. melanogaster* embryonic expression [40]. Fluorescence intensities were observed for 3,075,693 probes across 105,897,358 bp of the fly genome. Each of the oligos used in the array was 25bp in length, spaced at ~35bp intervals genome-wide. Unlike ESTs, which allowed us to assign TSS associations at the level of individual nucleotides, the limited tiling resolution restricted our ability to distinguish differences in transcriptional activity of promoters at individual TSS. Therefore, we analyzed the temporal embryonic utilization of peaked promoters separated by more than 100bp and broad promoters. Given this level of resolution, we needed to distinguish sites of true transcription initiation from background fluorescence noise. Transcription levels of internal coding regions of genes can be distinguished from background by large differences in fluorescence levels. However, boundaries between TSS locations and non-transcribed adjacent intergenic sequences are typically not as clear. Recent studies used a median estimator and the binomial distribution to distinguish expression from background [89-91]. These methods effectively identified positive expression of individual tiles; however, they did not take into account neighboring tiles to determine expression boundaries. Instead, we subtracted the difference in fluorescence intensity of several tiles upstream of TSS locations from those downstream to evaluate expression because we were interested in finding the boundaries of transcripts. TSSs may not be detected in this analysis if intensity differences fell below the specific thresholds, or if genes had exceptionally short first exons for which the tile size of 25bp is too large to obtain a reliable signal above the background.

The fluorescence intensities of random sites were used to estimate the rate of false positives. The number of 100bp sequences surrounding random sites that were deemed transcribed ranged from 92 to 162 across all 12 periods, resulting in a low expected false positive rate of .02 to .035 (Table S4). This agrees with the .02-.046 rate of transcription previously observed in intergenic regions across the 12 periods [40]. The true false positive rate may be lower than this approximation as some random intergenic locations may correspond to unidentified exons, or the expression of other genomic elements. In comparison, Manak et.al. [40] approached promoter utilization for TSSs from the same tiling data computationally by first clustering transfrags and manually curating 5’ start sites. Two disadvantages of this approach are lower TSS resolution in identifying TSS locations, and lower accuracy of clustering assignments. The average and median lengths of the transfrags used were 328 and 197bp, respectively, which corresponds to at least two non-overlapping core promoters, with additional sequence in between. Their clustering strategy had a reported accuracy of 65-77% when the number of correctly identified new first exons of known genes from the tile fluorescence intensities were evaluated by sequencing.

| Time Point | Hours | Difference Thres. | # False Positives | False Positive Rate |
| --- | --- | --- | --- | --- |
| 1 | 0-2 | 36.5 | 103 | 0.022 |
| 2 | 2-4 | 23.5 | 94 | 0.020 |
| 3 | 4-6 | 21.0 | 158 | 0.034 |
| 4 | 6-8 | 23.5 | 121 | 0.026 |
| 5 | 8-10 | 29.5 | 122 | 0.026 |
| 6 | 10-12 | 35.0 | 126 | 0.027 |
| 7 | 12-14 | 24.5 | 113 | 0.024 |
| 8 | 14-16 | 32.0 | 162 | 0.035 |
| 9 | 16-18 | 28.0 | 113 | 0.024 |
| 10 | 18-20 | 21.0 | 149 | 0.032 |
| 11 | 20-22 | 23.0 | 92 | 0.020 |
| 12 | 22-24 | 18.5 | 93 | 0.020 |

Table S4

When pooling both types of promoters together and comparing their utilization to the EST associations, there were 2,558 sites with embryo EST associations, and 2,106 without. On average, there were 1.6 times more sites with embryo EST associations detected as transcribed by the tiling array at each time point than those with non-embryo EST associations (head-specific, testes-supported, etc) (Figure 7A). When normalized by the total number of sites with embryo and non-embryo EST associations, this is a statistically significant trend (p < .01, paired t-test). As both experiments were performed on the embryo, this confirms that measurements of transcript expression are consistent across data types. There were 68.4% of sites with embryo associations and 46.9% of sites with non-embryo EST associations whose promoters were utilized in at least one of the 12 2 hour periods, as determined by the tiling array. The latter group contained sites with “mixed” associations, many of which are from the Exelexis EK library, which was generated from embryos, imaginal discs, and adult heads [76]. Thus, active initiation sites with non-embryo EST associations most likely included some that are, in fact, active in embryonic transcription programs. The difference between the utilization of promoters with tiling support and those with and without embryo EST associations is greatest during the first two time points, decreases throughout the remaining time points, and disappears at time point 12 (Figure 7A).

The time course tiling data allowed us to consider temporal patterns of promoter activity in greater detail than the EST conditions. The most frequent patterns for all promoters (peaked and broad) were “all off”, i.e. no utilization during any period (41%), and “all on”, i.e. expression for the entire 24-hour duration of embryogenesis (5.8%; 272 TSSs). Patterns observed for more than five promoters are listed in Additional data file 6. There was a tendency for more frequent patterns to show expression at contiguous time points. This provides support that these patterns are not artifacts of the tiling array processing, but rather, true promoter utilization over longer time intervals. Some patterns showed expression for more than one contiguous time point. This may result from not detecting transcription for cases in which the expression level fell just below the determined threshold. The pattern may also reflect the utilization of more than one promoter that we were unable to differentiate due to the resolution of the tiling array. For instance, a common pattern had activity at four time points: T,T,N,N,T,T,N,N,N,N,N,N (T=transcribed; N=non-transcribed). For overlapping promoters in a TSS cluster group, this pattern may be the result of the combination of utilization of one promoter for a maternally inherited transcript during periods 1 and 2, and another promoter utilized for early zygotic transcription during periods 5 and 6.

The onset and duration of expression patterns across the 12 time points in the tiling array corresponded to three broad transcriptional cycles: 1-2, 4-8, 9-12 noted above and by Manak et al. The most common contiguous patterns tended to start and/or stop at cycle boundaries. The first and second 2 hour periods were assumed to correspond to transcripts that were maternally inherited, periods 4-8 covered early zygotic transcription, and periods 9-12 signified late zygotic transcription. As noted, the enrichment for the third cycle was smaller and less defined than for the first two. There were 141 genes whose alternative TSSs were more than 100bp apart and had the same EST condition associations. For 26 of these genes, no promoters were active at any time point. Only five genes had promoters with the same activity pattern, and all five showed activity across all 12 time points. The remaining 110 (78%) genes had different temporal patterns of utilization across alternative promoters. This clearly demonstrates that while associations may be the same across larger global conditions, such as those corresponding to the EST libraries, data on a more precise scale may reveal differences in initiation patterns.

**Motif Presence and Associations to Spatiotemporal Conditions**

Overall, individual motifs had a much higher frequency in both peaked and broad *Drosophila* core promoters than observed collectively as part of motif modules. This may be evidence of motifs functioning independently of each other, in spite of their ability to synergistically cooperate. It may also suggest that individual motifs can have a general role in the binding of transcription factors to increase the overall rate of initiation, even though they may have a more restricted function when present in specific modules. Furthermore, as different repertoires of transcription factors are present under varying conditions, dual roles of motifs may correspond to different conditions. For instance, the TF binding to the DRE may be present individually in one condition, resulting in the DRE generally increasing the rate of transcription, while both TFs binding to the DRE and Ohler 7 may be present in a second condition, allowing for the complex to be more restrictive in interactions to recruit RNA pol II to the DNA.

In the association of motif frequencies to EST conditions, the eight elements and their modules were identified in the promoter regions as described (see Materials and Methods). Broad promoters were excluded from this analysis because each of the TSSs in the TSS cluster groups could have a different EST association, and due to the weak spatial biases of some motifs, distinct assignments of elements to conditions would be difficult for overlapping core promoters. In both sets, the TATA, INR, DPE, and MTE had higher probabilities of occurring in their preferred windows than the Ohler 1, DRE, Ohler 6, and Ohler 7 elements. This mirrored the higher enrichment signal for the location-specific motifs. However, associations between either set of motifs to the EST conditions were not found to be significant, and therefore, not investigated further.

As described in the main text, significant motif associations were found for both maternal and zygotic genes as determined by the tiling array analysis. In the set of peaked promoters with both maternal and zygotic utilization, slight fluctuations in element frequencies of the TATA, MTE, Ohler 1, and Ohler 7 were seen, however, the differences were not large enough to alter their overall pattern that mirrored that of maternal utilization. There were three times more sites with utilization during both phases of embryogenesis containing the Ohler 1, DRE, Ohler 6, and Ohler 7 motifs, than those having maternal utilization alone. When the sequence elements were restricted to their preferred windows in the peaked core promoters, the same trends of maternal versus zygotic element preference were observed (Figure S3B). Similar results were seen for broad promoters (data not shown). In both promoter types, the TATA and INR had the highest motif frequencies, and the Ohler 6 and Ohler 7 had the lowest. This confirms the importance of location for the TATA and INR, and the presence of the Ohler 6 and Ohler 7 motifs throughout promoters utilized both maternally and zygotically.

**Downstream Effects of Alternative TSSs**

While we did not explore this in the current study, alternative promoters may have downstream effects: the diversification of the gene’s isoforms, an increase in the complexity of the gene’s architecture and possibly, an expansion of the biochemical role of the gene’s function. Analysis on full-length mouse cDNAs has shown a significantly higher correlation between the utilization of alternative promoters and the occurrence of alternative splice isoforms and multiple start codons, than for genes with one promoter [92]. The association of alternative promoters to alternative transcript isoforms may simply result from the condition-specific expression of transcription and splice factors that independently lead to diversified isoforms. A more intriguing model suggests a direct link between the preferential recruitment of splicing factors to mRNAs transcribed from alternative promoters and the recruitment of splicing factors by condition and promoter-specific transcription factors [93-95]. This could result from the usage of multiple promoters always generating different 5’UTRs sequences, which are known to harbor functional elements. A third model suggests that alternative promoters have more subtle effects, such as affecting the mRNA’s half-life, rate of translation, localization, or overall level of protein production.


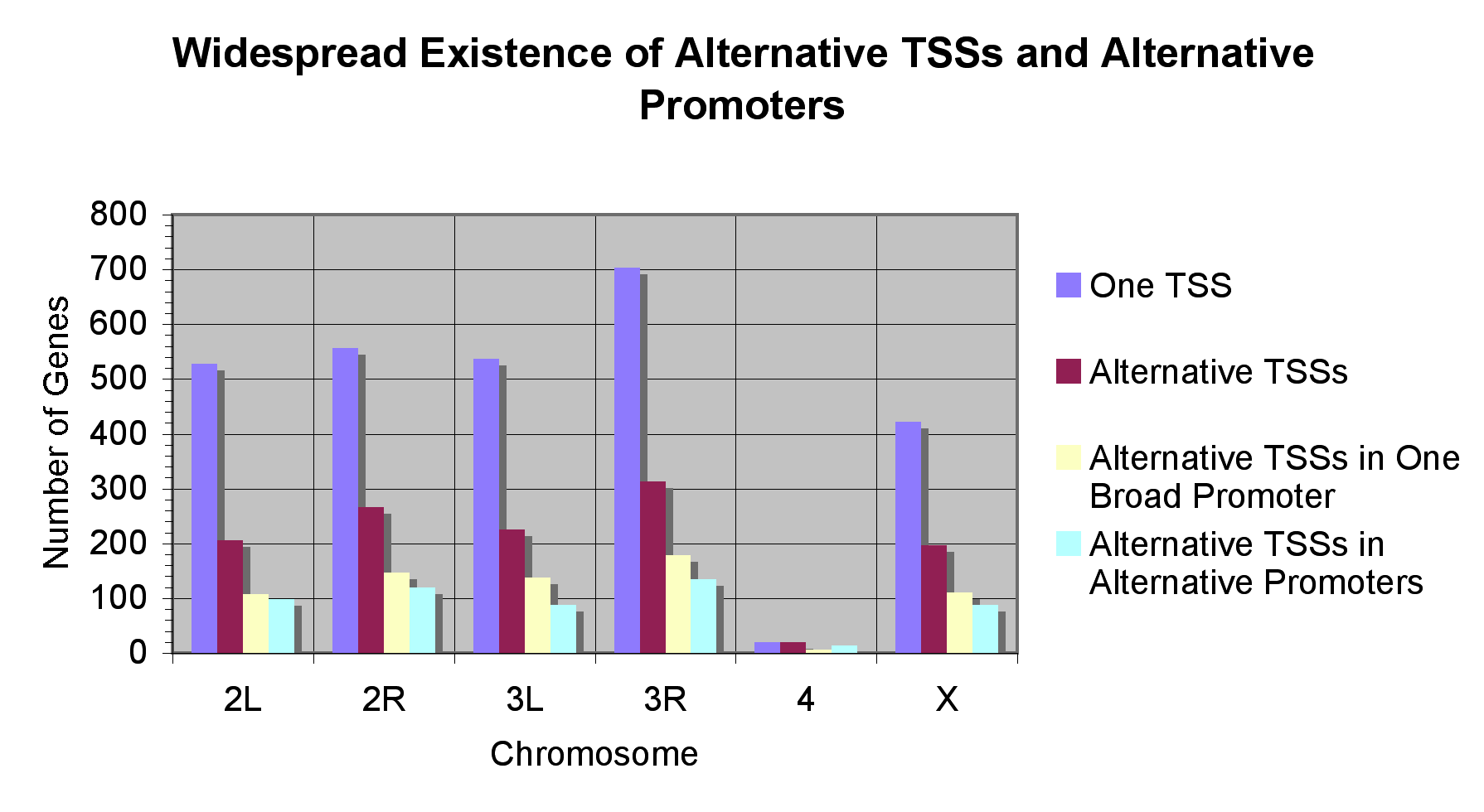


Figure S1


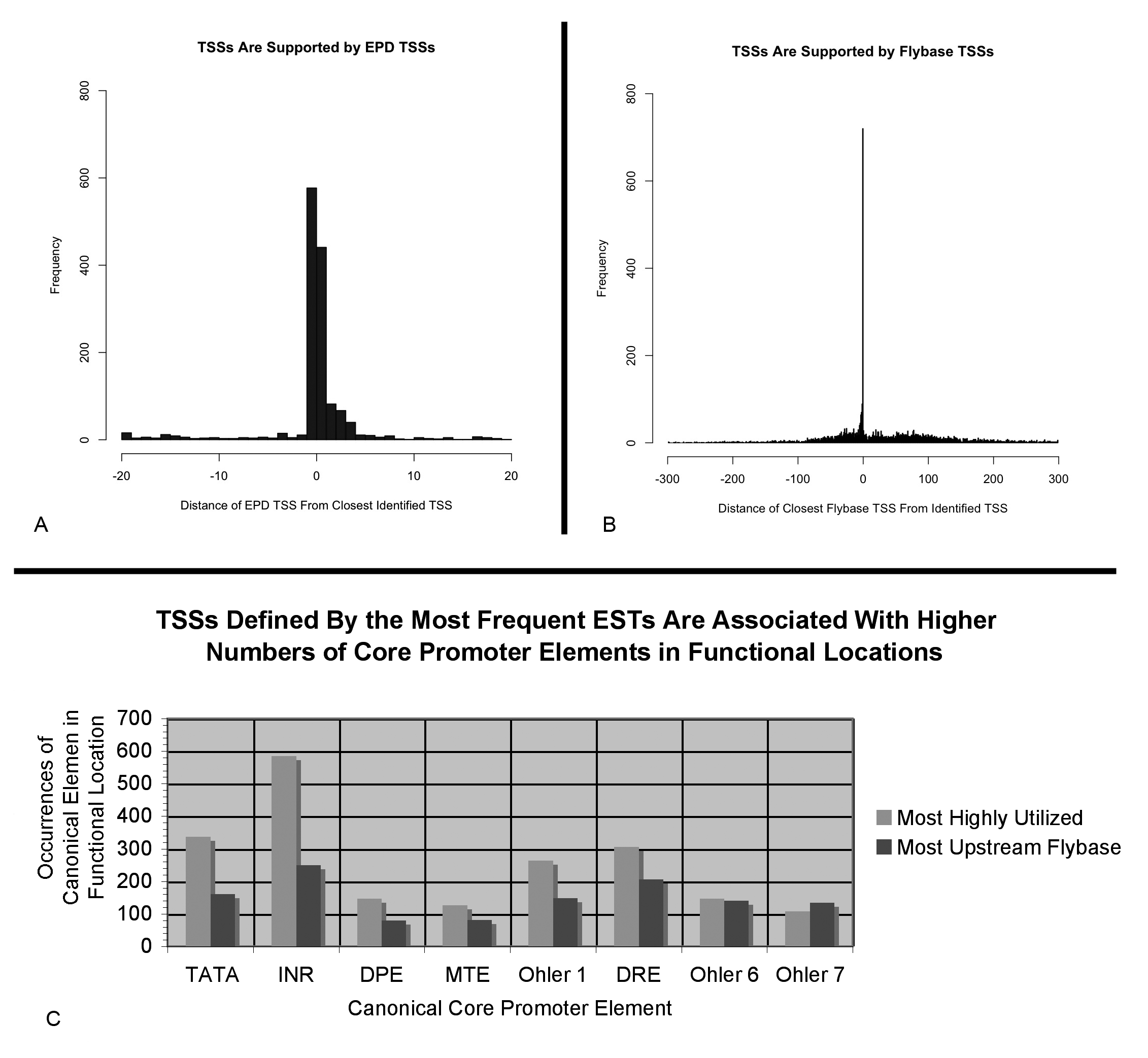


Figure S2


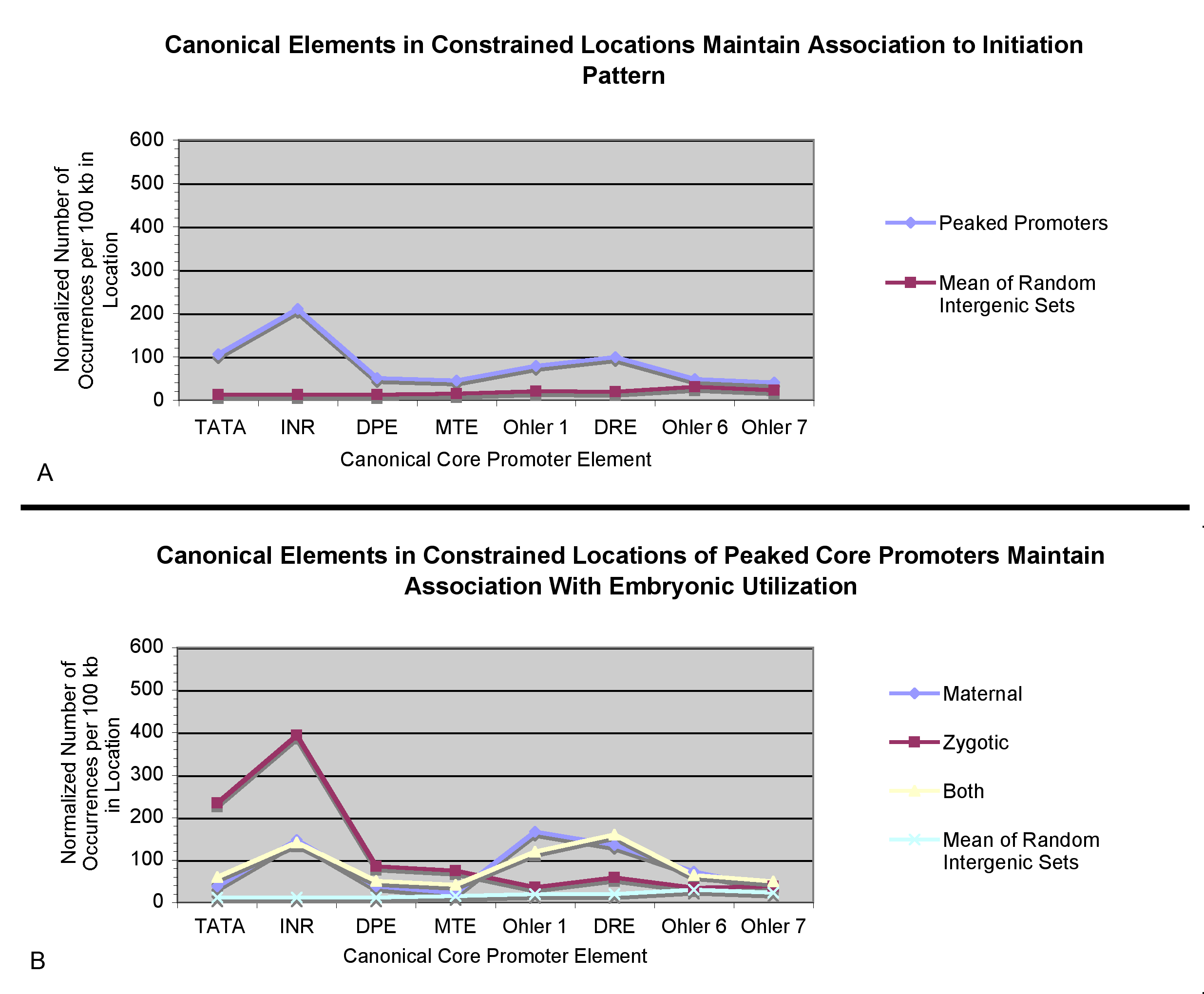


Figure S3


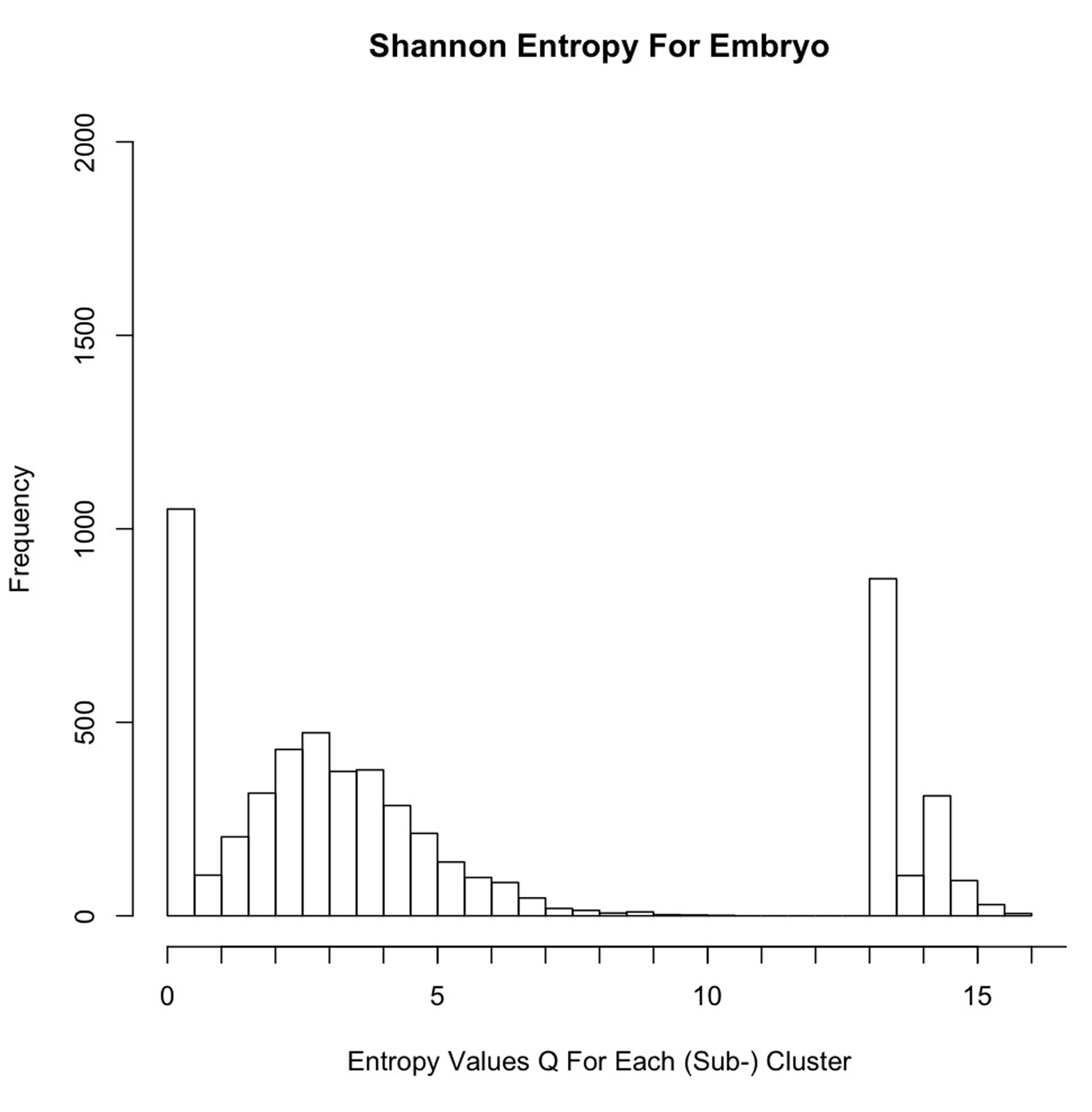


Figure S4
